# Optimization of p-SnS/n-CdS heterojunction solar cells *via* impedance spectroscopy and SCAPS modeling: impact of doping, thickness, and series resistance

**DOI:** 10.1039/d5ra09139j

**Published:** 2026-02-18

**Authors:** Rkia Zari, Benjamin K. Korir, Nassima Riouchi, Taoufik Garmim, Abderrahmane Elmelouky, Elmesbahi Elmostafa, Mohammed Salah, Joshua K. Kibet

**Affiliations:** a Innovative Materials, Energy and Sustainable Development Laboratory, Faculty of Sciences and Technology, Cadi Ayyad University Marrakech Morocco; b Department of Chemistry, Egerton University P. O. Box 536 Egerton 20115 Kenya jkibet@egerton.ac.ke; c LCM2E, Laboratory of Molecular Chemistry, Materials and Environment, Multidisciplinary Faculty of Nador (FPN), Mohammed Premier University B. P. 300 Selouane Nador 62700 Morocco; d Interdisciplinary Laboratory for Fundamental and Applied Science, Higher Normal School, Hassan II University Casablanca Morocco; e Department of Physic Condensed Matter Laboratory, Faculty of Sciences, Chouaib Doukkali University El-Jadida Morocco; f Department of Physics, Chouaib Doukkali University El Jadida Casablanca-Settat Morocco; g Molecular Modeling and Spectroscopy Research Team, Department of Chemistry, Faculty of Sciences, Chouaïb Doukkali University El Jadida Morocco

## Abstract

This work presents a detailed study of p-SnS/n-CdS heterojunction solar cells with Al–ZnO/i-ZnO window layers, combining simulations with the one-dimensional solar cell capacitance simulator (SCAPS-1D) and impedance spectroscopy for an in-depth investigation of the mechanisms limiting device performance. The effects of key cell parameters, such as absorber layer thickness, doping concentration, series resistance (*R*_s_), and operating temperature, were systematically explored, as these factors strongly influence solar cell performance. Optimal efficiency was achieved with a 4 µm SnS absorber layer, resulting in a power conversion efficiency (PCE) of 22.76% and an open-circuit voltage (*V*_oc_) of 0.77 V under standard illumination conditions. Although increasing the *R*_s_ significantly degraded the fill factor (FF) and PCE, *V*_oc_ and short-current density (*J*_sc_) remained largely stable. The utility of complex impedance proved crucial in understanding the underlying physical mechanisms of each parameter (thickness, doping, *R*_s_) and temperature, and their influence on overall efficiency. In the 0.1 Hz–1 GHz frequency range, two relaxation processes were revealed: a low-frequency response attributed to bulk recombination at the CdS/SnS interface, and a high-frequency response associated with interfacial polarization within the ZnO layers. Notably, the ZnO/CdS and CdS/SnS interfaces exhibited opposing thermal trends, reflected by the evolution of the relaxation times. The coupling between SCAPS-1D and the dynamic study *via* impedance spectroscopy highlights the importance of absorber doping, optimized thickness and minimized *R*_s_ as key parameters for obtaining high-efficiency SnS-based thin-film photovoltaic cells, and provides essential information on the interfacial dynamics and volumetric recombination processes that govern device performance and stability.

## Abstract 2

This work presents a detailed study of p-SnS/n-CdS heterojunction solar cells with Al–ZnO/i-ZnO window layers, combining numerical simulations with impedance spectroscopy for an in-depth investigation of the mechanisms limiting the performance of these solar cells.

## Graphical abstract

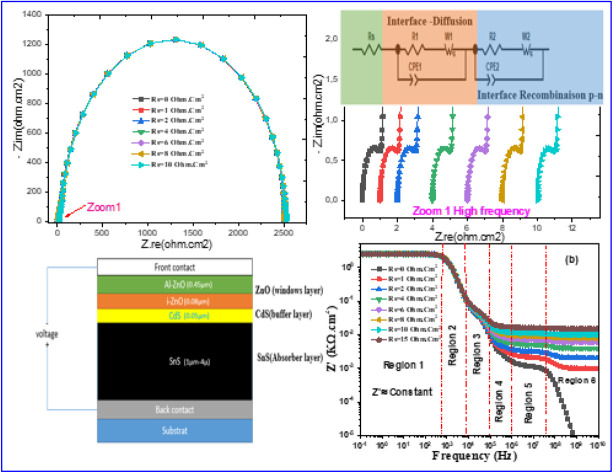

## 1 Introduction

The field of renewable energy has attracted significant scientific attention, with particular emphasis on the development of photovoltaic technologies. Among the photovoltaic technologies, thin-film solar cells have emerged as promising candidates due to their potential for high efficiency and low fabrication cost. Well-known examples include CdTe and CIGS absorbers, which have reached certified efficiencies of 19.6% and 20.4%, respectively.^1,2^ These achievements have stimulated interest in the exploration of alternative polycrystalline materials. However, the toxicity of cadmium in CdTe and the scarcity of indium and gallium in CIGS raise environmental and economic concerns, motivating the search for sustainable and earth-abundant alternatives.^3,4^ In this context, Cu_2_ZnSnS_4_ (CZTS) and tin sulphide (SnS) have emerged as promising absorber materials. SnS is a IV–VI group p-type semiconductor^5^ characterized by a high absorption coefficient (>10^4^ cm^−1^)^6^ and a direct bandgap of ∼1.3 eV,^7^ which matches well with the solar spectrum. These properties, together with its abundance, non-toxicity, and low cost, make SnS a promising material for thin-film solar cells, with theoretical efficiency limits approaching 32%.^5,8^ To further enhance device performance, a detailed investigation of the electrical properties through resistance (*R*), dielectric behavior through capacitance (*C*), and charge transport mechanisms in heterojunction solar cells is required.^9^ Impedance spectroscopy (IS) has emerged as a versatile analytical technique, extensively employed in the characterization of organic solar cells, quaternary inorganic CZTS devices,^10^ and CdTe-based solar cells,^11,12^ as well as perovskite systems^13^ and doping-related investigations in CIGS technologies.^14^ IS provides comprehensive and efficient insight into recombination mechanisms, charge carrier transport, and interface dynamics. Recent studies have highlighted its importance in probing electrochemical and electronic processes in solar cells, establishing IS as a suitable and effective tool for the analysis of SnS-based heterostructures.^15,16^

Scientific studies on SCAPS-1D (one-dimensional solar cell capacitance simulator) simulations and careful material engineering have demonstrated that device efficiency can be dramatically improved by minimizing recombination losses.^17^ The growing momentum toward sustainable solar technologies has led to the emergence of lead-free and halide-free perovskites.^18,19^ Concurrently, fundamental studies are ongoing on how device performance can be enhanced by improving interface quality, optimizing device thickness, and adjusting doping concentrations, which directly influence device performance.^20^ In addition to novel materials science developments, governance frameworks are striving to ensure that emerging technological advances translate into real-world carbon reductions.^20^ As solar power increasingly attracts applications in smart grids, biomedical systems, and the Internet of Things (IoT), there is a need to ensure that they become reliable and secure.^19^ Network-level protection through IoT sensors and material-level optimizations has benefited from advances in deep and machine learning. Despite significant advances in heterojunction and thin-film solar cells, the combined influence of absorber thickness, doping concentration, and p-SnS/n-CdS doping concentration remains underexplored. In addition, few studies have provided comprehensive SCAPS modelling coupled with impedance spectroscopy to investigate how these parameters influence recombination, charge transport, and overall efficiency. This study systematically models a SnS/CdS heterojunction which includes non-toxic and earth-abundant materials for potentially scalable solar applications. Remarkably, improved understanding of the fundamental knowledge relating to the SnS/CdS heterojunction provides a roadmap for improving the efficiency of these viable candidates.

Towards this end, the aim of this work is to perform a comprehensive analysis of Al–ZnO/i-ZnO/CdS/SnS heterojunction solar cells by combining SCAPS-1D simulations and impedance spectroscopy (IS). The study focuses on three key aspects: (i) the effect of absorber thickness and *R*_s_ on the photovoltaic parameters (*η*, FF, *J*_sc_, and *V*_oc_), (ii) the influence of operating temperature (300–400 K) on device performance and stability, and (iii) the identification of relaxation processes and interface properties through IS in the frequency range 0.1 Hz–1 GHz. By correlating *J*–*V* analysis with impedance deconvolution, this work provides new insights into charge transport, recombination, and interfacial phenomena, contributing to the optimization of SnS-based thin-film solar cells for stable high-efficiency operation.^21–25^

## 2 Simulation methodology

The numerical modeling of SnS/CdS/i-ZnO/Al–ZnO thin-film solar cell was performed using SCAPS-1D.^5,26^ This device simulator solves semiconductor equations including Poisson (eqn (1)) and continuity equations (eqn (1) and (2)), while considering the Shockley–Read–Hall (SRH) recombination mechanisms.^27,28^ The model examines layer properties, defect states, and optical generation under one sun at 1000 W m^−2^ and AM 1.5G.^29^ The interface defect densities at the CdS/SnS and CdS/ZnO interfaces in the SCAPS simulations were selected based on a combination of reported values in the literature for similar heterojunctions and a calibration process to ensure physical consistency with experimentally achievable device behaviour. In particular, initial ranges of interface defect densities (10^10^ to 10^13^ cm^−2^) were considered, which are commonly used for chalcogenide and oxide interfaces in SCAPS-based studies. SCAPS-1D thus provides an efficient platform for optimizing SnS-based photovoltaic devices and interpreting impedance spectroscopy data.^30–32^1
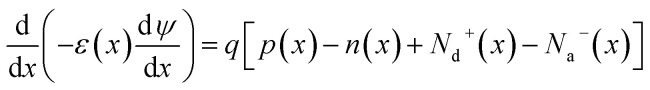
Here, *ε* denotes the permittivity denotes the electron charge, *ψ* denotes the electrostatic potential, *n* is the total electron density, *p* represents the total hole density, *N*_d_^+^ represents the ionized donor-like doping concentration, and *N*_a_^−^ denotes the ionized acceptor-like doping concentration. Continuity equations for electrons and holes, and drift-diffusion equations as shown in eqn (2).^33,34^2
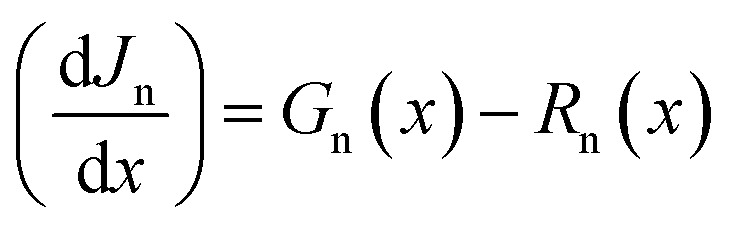
3
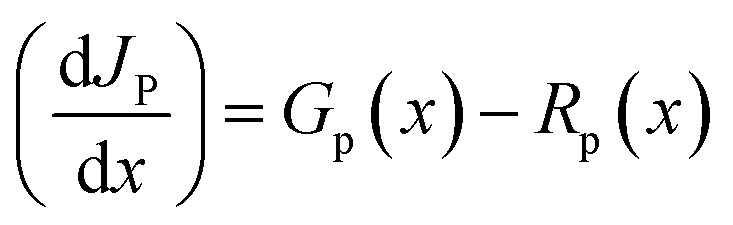
*G*_p_, and *G*_n_ is the generation rate of holes and electrons distribution respectively, *R*_n_ and *R*_p_ are electrons and holes recombination rate respectively. *J*_n_ and *J*_p_ are electrons and holes current density respectively.^35^

Further, SCAPS-1D incorporates various recombination mechanisms that influence solar cell performance.^36^ By defining these mechanisms, researchers can better understand and mitigate energy losses within the device. Another important feature of SCAPS-1D is its ability to model defect states both within the bulk material and at interfaces. Parameters such as defect energy levels, capture cross-sections, and defect densities can be customized, enabling the study of how these imperfections affect exciton dynamics and overall device performance. Furthermore, appropriate electrical boundary conditions were applied at both the front and back contacts through the contact work function and the surface recombination velocities (SRV) for electrons and holes. The applied parameters are summarized in Table 1. At the front contact, work functions of 4.69 eV and 4.34 eV were used for SnS, in order to form quasi-ohmic contacts for electron extraction. For both interfaces, the surface recombination velocities for electrons and holes were set to *S*_e_ = *S*_h_ = 1 × 10^7^ cm s^−1^, representing realistic, non-ideal contact conditions, as summarized in Table 1.

**Table 1:** Material parameters for front and back contact of the simulated model solar cell structure

Parameter	Front contact	Front contact
*φ* (eV)	4.69	4.34
*S*_e_ (cm s^−1^)	1 × 10^7^	1 × 10^7^
*S*_h_ (cm s^−1^)	1 × 10^7^	1 × 10^7^

Further to optical and electrical modelling, SCAPS-1D supports bias condition simulations, including voltage sweep and capacitance–voltage (*C*–*V*) analysis.^37^ The voltage sweep function is essential for generating current–voltage (*J*–*V*) characteristics, which are crucial for determining key performance metrics such as power conversion efficiency PCE, FF, *J*_sc_, and *V*_oc_. To evaluate the robustness of our results, a sensitivity analysis was performed by systematically varying the interface defect densities over several orders of magnitude while keeping all other parameters constant. The influence on the main photovoltaic outputs (*V*_oc_, FF, *R*_s_, and *R*_ct_) was then examined. Meanwhile, *C*–*V* analysis helps investigate doping profiles and defect states, providing deeper insights into the electrical behaviour of the solar cell.^37^ Overall, SCAPS-1D is a powerful and user-friendly simulation tool that offers detailed insights into the electrical and optical behaviour of solar cells. Despite some limitations, its versatility and ease of use make it an essential resource for optimizing solar cell performance and guiding experimental research.

**Fig. 1:**
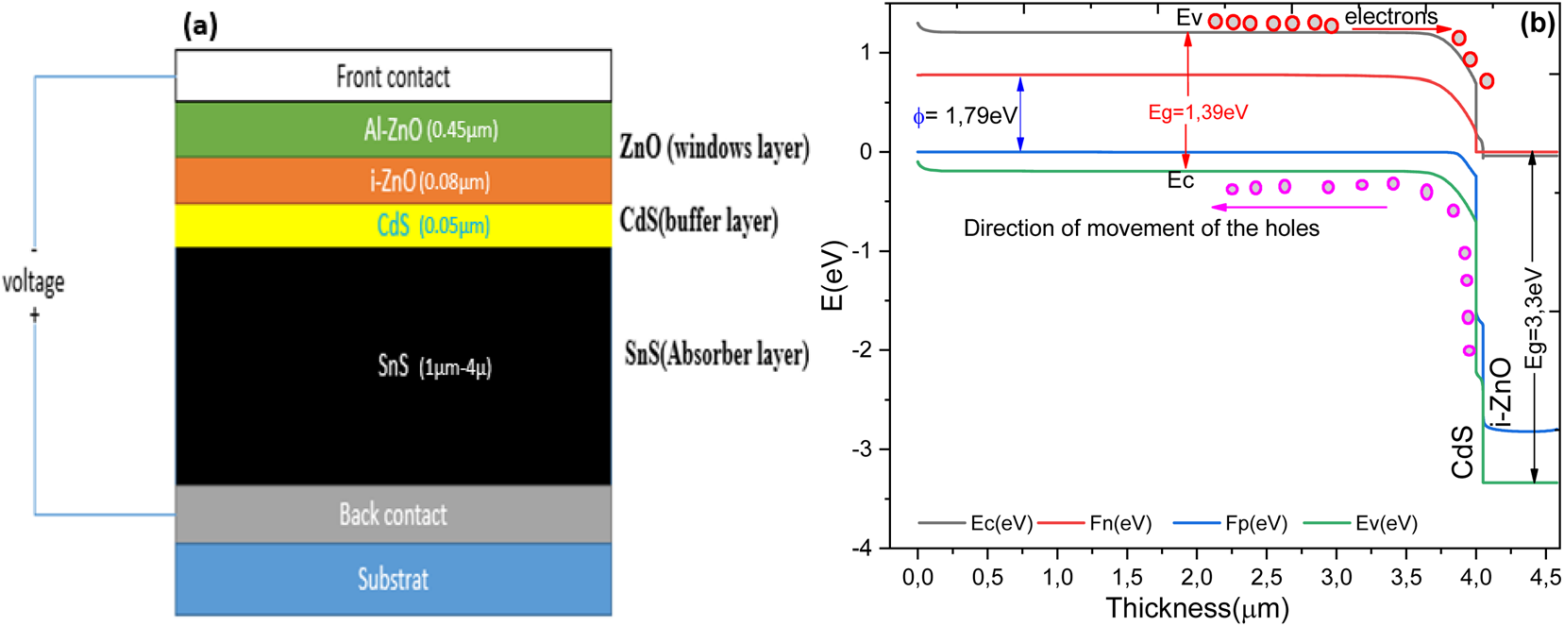
The proposed solar cell's structure (a) and (b) corresponding energy levels band diagram.

Fig. 1a presents the solar cell structure whereas Fig. 1b provides an overview of the energy levels within the proposed photovoltaic cell. This diagram is essential for understanding the electronic processes that govern charge carrier transport, a key mechanism in the device's operation. At the core of this diagram are the conduction band (CB) and the valence band (*V*_B_). The conduction band represents the range of energy levels where electrons can move freely, enabling electrical conduction. In contrast, the valence band consists of lower energy levels where electrons remain bound to atoms and do not contribute to conduction.^38^ The energy gap between these two bands is a fundamental parameter that defines the electronic properties of the material. Table 2 presents the basic input parameters used in this work. These parameters were obtained from experimental theoretical studies in literature to ensure the simulated data is validated. Further, the thickness of various layer thicknesses was optimized for better electrical output parameters.

**Table 2:** Parameters obtained by simulation SCAPS-1D

Layers	Al–ZnO	i-ZnO	CdS	SnS
Thickness (µm)	0.4	0.08	0.05	1–5
Bandgap (eV)	3.3	3.3	2.41	1.4 (ref. 39)
Electron affinity (eV)	4.4	4.4	4.5	4 (ref. 40)
Dielectric permittivity	9	9	9	12.5
CB effective density of states (cm^−3^)	2.2 × 10^19^	2.2 × 10^19^	1.8 × 10^19^	7.5 × 10^18^
*V*_B_ effective density of states (cm^−3^)	2.2 × 10^19^	2.2 × 10^19^	2.4 × 10^19^	1 × 10^19^
Electron thermal velocity (cm s^−1^)	1 × 10^7^	1 × 10^7^	1 × 10^7^	1 × 10^7^
Hole thermal velocity (cm s^−1^)	1 × 10^7^	1 × 10^7^	1 × 10^7^	1 × 10^7^
Electron mobility (cm^2^ V^−1^ s^−1^)	100	100	35	100
Hole mobility (cm^2^ V^−1^ s^−1^)	25	25	50	4
Shallow uniform donor density *N*_D_ (cm^−3^)	1 × 10^20^	1 × 10^20^	1 × 10^17^	0
Shallow uniform acceptor density *N*_A_ (cm^−3^)	0	0	0	5.7 × 10^15^
Ref.	41	42	41	43

## 3 Results and discussion

### 3.1 Electrical behavior of ZnO/CdS/SnS structure

#### 3.1.1 Effect of SnS absorber thickness on *J*–*V* characteristics

**Fig. 2:**
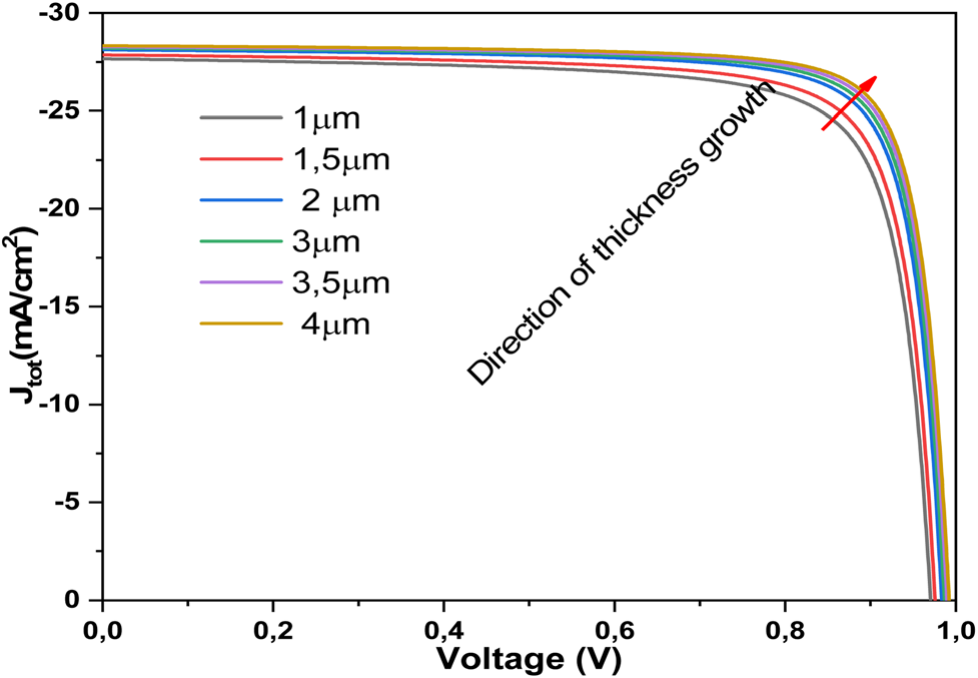
Current–voltage curves at various absorber thicknesses.

The current–voltage characteristics as a function of temperature and absorber layer thickness, within a temperature range of 300 K to 400 K, as well as the influence of *R*_s_ were analyzed *via* SCAPS-1D. The effect of the SnS absorber layer thickness was studied for various values between 1 µm and 4 µm, as illustrated in Fig. 2. Increasing the absorber thickness improves light absorption and photocurrent generation, thus leading to an increase in the short-circuit current density (*J*_sc_). However, beyond an optimal range of around 3 to 5 µm, further increases in thickness degrade cell performance due to increased Joule losses at the p–n junction.

**Fig. 3:**
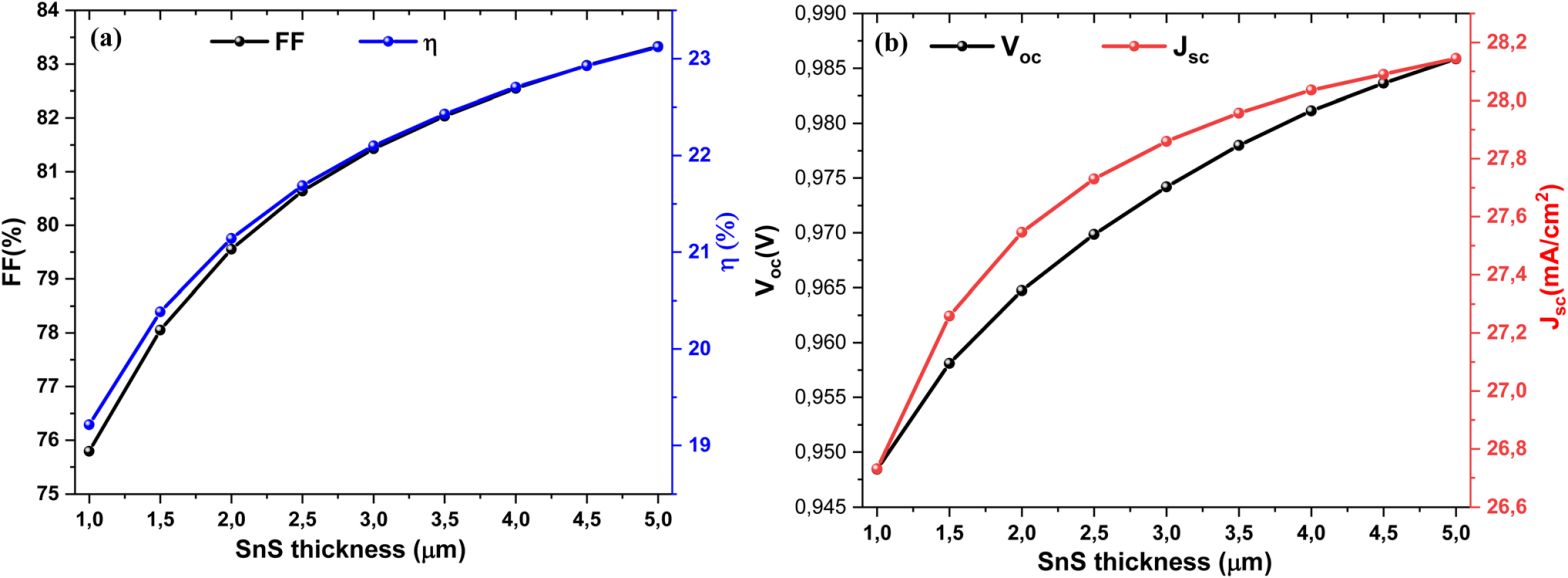
Dependence of (a) FF and efficiency (*η*), and (b) *V*_oc_ and *J*_sc_ on the SnS layer thickness.

The impact of absorber thickness on the performance metrics of the cell structure are presented in Fig. 3. It is observed that *J*_sc_ and *V*_oc_ increase significantly with increasing absorber layer thickness. This is explained by more efficient absorption of longer-wavelength photons when the absorber is thicker, leading to increased electron–hole pair generation. This increase results in improved *J*_sc_ and *V*_oc_, and consequently, improved PCE. An increase in FF is also observed. However, excessive absorber layer thickness can lead to resistive effects that may degrade the form factor.^44^

#### 3.1.2 Nyquist plot measurements for initial and optimized solar cell devices

**Fig. 4:**
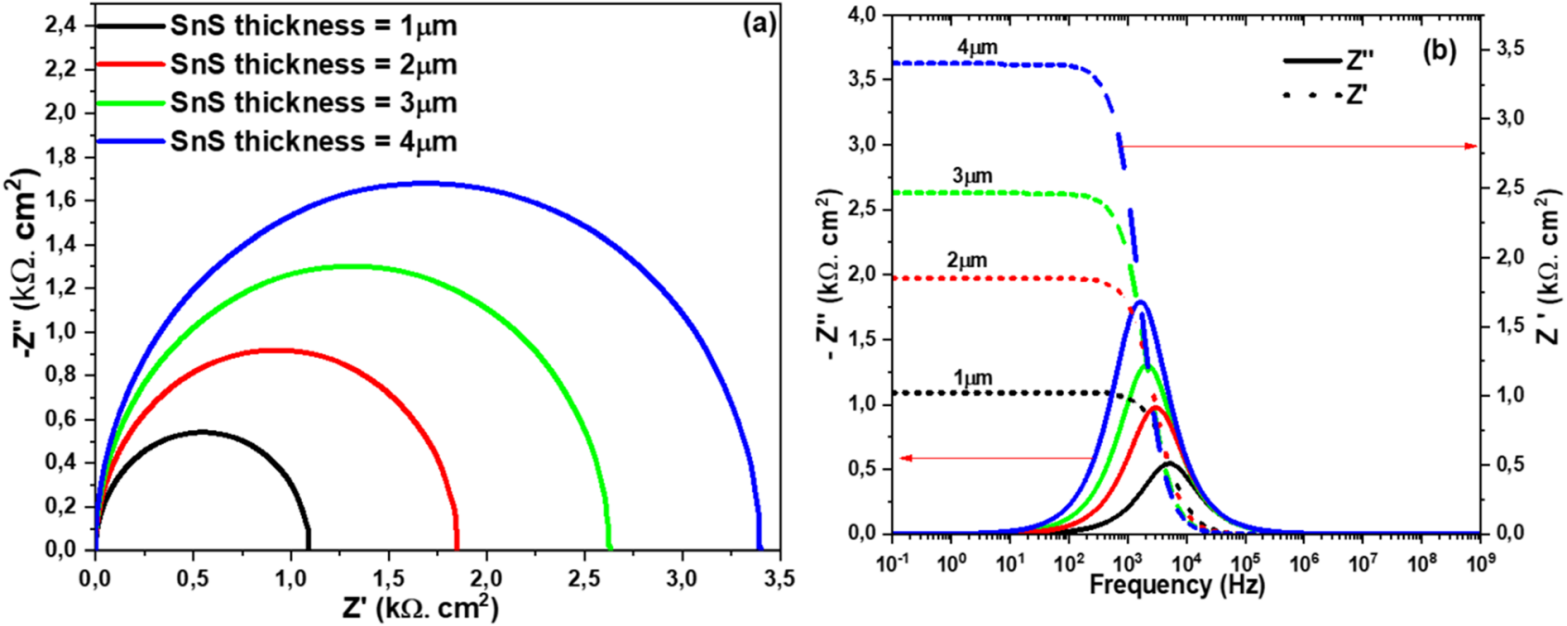
Nyquist diagram (a) and (b) *Z*″ and *Z*′ dependence on frequency at different SnS thicknesses.

Impedance spectroscopy is a powerful tool for investigating solar cells, as it provides valuable insights into charge carrier transport and the underlying operational mechanisms.^45^ In this context, Nyquist plots derived from capacitance–frequency (*C*–*F*) measurements effectively illustrate these behaviors, complemented by Bode plots for a more comprehensive analysis. Fig. 4a clearly shows the variation of the imaginary part as a function of the real part of the complex impedance. This Nyquist plot highlights the influence of the absorber thickness on the arc diameter, which increases as the thickness increases. Fig. 4a clearly illustrates the relationship between the imaginary and real components of the complex impedance. The Nyquist plot reveals that the arc diameter increases with increasing absorber thickness, highlighting its influence on the impedance response.

### 3.2 Effect of *R*_s_ for SnS absorber thickness = 4 µm at *T* = 300 K

#### 3.2.1 Current voltage simulation at various *R*_s_ values

**Fig. 5:**
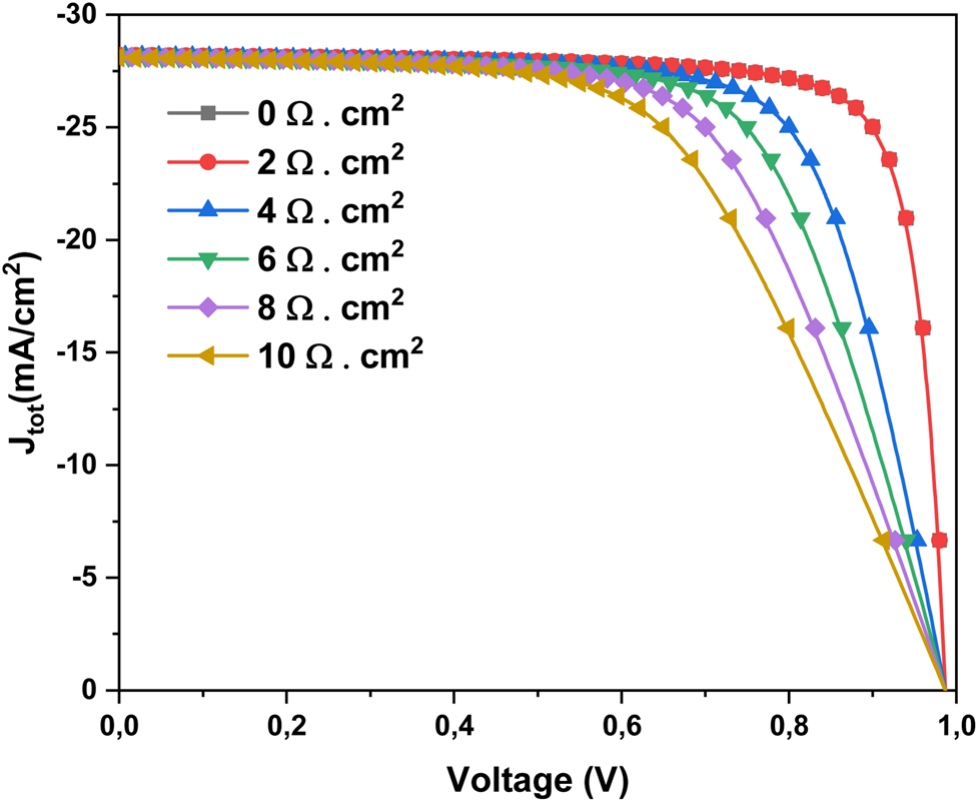
Current density *versus* voltage measurements at different *R*_s_ values.

Following the optimization of the solar cell through fine-tuning the SnS absorber layer thickness, the impact of *R*_s_ on the device performance is further explored using electrochemical impedance spectroscopy (EIS). The analysis of both Nyquist and Bode plots enables a comprehensive evaluation of the cell's electrical and dielectric behavior, offering valuable insights into charge transport and recombination mechanisms, which are critical for high-efficiency operation.^46^ Furthermore, a preliminary investigation of the current density as a function of *V*_oc_ is carried out to determine the *V*_oc_ corresponding to the maximum power point (MPP), and to refine additional performance parameters such as FF and output power.^46,47^Fig. 5 shows *J*–*V* characteristics of Al–ZnO/i-ZnO/CdS/SnS solar cells for different *R*_s_ values (*R*_s_ = 0–10 Ω cm^2^). At low *R*_s_, the device exhibits sharp diode behavior with high FF and efficiency. As *R*_s_ increases, resistive losses become dominant, reducing the slope near *V*_oc_ and leading to lower FF and *η*. The short-circuit current density (*J*_sc_) remains relatively unchanged, while *V*_oc_ decreases slightly at higher *R*_s_ values.

According to the trends shown in Fig. 7a and b, it is clear that an increase in *R*_s_ significantly affects the overall performance of the photovoltaic cell. This increase results in a progressive reduction in the FF, power conversion efficiency (*η*), *V*_oc_, as well as the total current density *J*_tot_. These observations are consistent with recent literature findings. For instance, Heredia-Rios *et al.*^48^ demonstrated that, in thin-film solar cells, *R*_s_ leads to ohmic losses that reduce the power extracted at the maximum power point. Similarly, Hosen *et al.*^49^ noted that high *R*_s_ distorts the *I*–*V* curve, reducing the effective area under the curve and thereby lowering the FF.

**Fig. 6:**
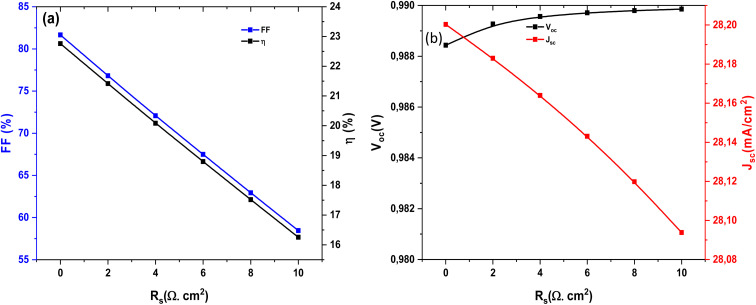
Variation of (a) FF and (*η*), (b) *V*_oc_ and *J*_sc_ parameters as a function of *R*_s_.

**Fig. 7:**
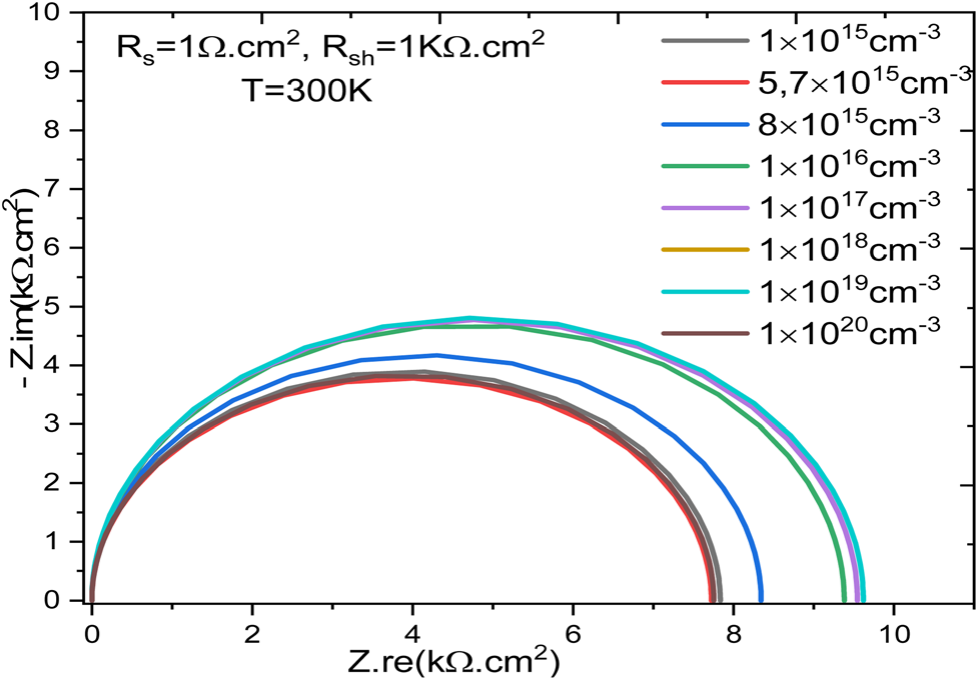
Nyquist plots for different doping concentrations at 300 K.

Regarding the *V*_oc_, it is generally less affected by *R*_s_ than other parameters. However, several studies^50^ have shown that at high *R*_s_ values, a slight decrease in *V*_oc_ can be observed, particularly if this resistance disrupts the balance between carrier generation and recombination. Finally, the total current density decreases mainly due to internal voltage drops, which limit the efficient transport of charge carriers across the cell. These findings highlight the critical importance of controlling and minimizing *R*_s_ in photovoltaic cell design. Achieving high conversion efficiency requires maintaining *R*_s_ as low as possible, a conclusion supported by recent studies on SnS-, CZTSe-, and perovskite-based solar cells.^50,51^ As shown in Fig. 7a, both the FF and the efficiency *η* exhibit an almost linear decrease with increasing *R*_s_. When *R*_s_ increases from 0 to 10 Ω cm^2^, FF drops from ∼81% to ∼57%, while *η* decreases from ∼23% to ∼16%. This strong sensitivity highlights the detrimental impact of resistive losses on charge extraction and power conversion. In Fig. 6b, the *V*_oc_ remains nearly constant, showing only a negligible variation across the studied *R*_s_ range. In contrast, the *J*_sc_ decreases slightly as *R*_s_ increases, reflecting the effect of resistive barriers on carrier collection (Fig. 6a). These results confirm that the main role of *R*_s_ is to limit the FF and efficiency, rather than significantly altering *V*_oc_ and *J*_sc_.

We also notice that the change in the efficiency value is insignificant when the thickness varies from 2,8 to 5 µm; therefore, it is not necessary to produce SnS/CdS/ZnO solar cells with a very large thickness because there is a trade-off between cell efficiency and cost for mass production. Large thicknesses introduce resistive components, which will be shown in the analysis part by impedance spectroscopy.^52^ Note also that the change in the efficiency value is insignificant when the thickness varies from 2.8 to 5 µm; therefore, it is not necessary to produce SnS/CdS/ZnO solar cells with a very large thickness because there is a trade-off between cell efficiency and cost for mass production.

Table 3 presents the simulation results obtained using the SCAPS-1D software for different thicknesses of the SnS layer. The results show that the cell efficiency reaches a maximum value of 22.76%, corresponding to an optimal SnS layer thickness of 4 µm.

**Table 3:** Solar cell performance metrics at varying absorber layer thicknesses for the i-ZnO/CdS/SnS configuration

SnS thickness (µm)	*V*_oc_ (V)	*J*_sc_ (mA cm^−2^)	FF (%)	*η* (%)
1	0.95	26.75	74.29	19.00
2	0.97	27.64	78.34	21.05
3	0.98	28.00	80.36	22.09
4	0.98	28.20	81.65	22.76

Parasitic resistances have a significant impact on the FF of solar cells. These resistances include the *R*_s_ and the shunt resistance (*R*_sh_), both of which are unavoidable in practical photovoltaic devices. Ideally, a PV cell should exhibit zero *R*_s_ and infinite *R*_sh_. However, in real conditions, the presence of these parasitic resistances modifies the current–voltage characteristics of the device.

### 3.3 Complex impedance spectrum simulation at different concentration doping for SnS

The impedance analysis confirms that doping concentration plays a decisive role in controlling charge transport and recombination in SnS-based solar cells. The Nyquist plots in Fig. 7 demonstrate the influence of doping concentration on the impedance behavior of SnS-based solar cells at 300 K. At low doping levels, the larger semicircle diameter reflects a higher recombination resistance, consistent with limited free carrier density and inefficient charge transfer at the junction. As the doping concentration increases, the semicircle diameter decreases, indicating enhanced conductivity and improved charge extraction. This behavior aligns with earlier reports on CdTe and CIGS thin-film devices, where controlled doping of the absorber was shown to reduce bulk resistive losses and improve junction quality.^53^

The effect of the donor doping concentration (*N*_D_) of the SnS absorber on the cell's electrical parameters was analyzed through the recombination resistance (*R*_rec_) in the Nyquist plot, *V*_oc_, and the FF. Increasing *N*_D_ leads to a significant change in the internal electric field within the junction, which directly influences the charge carrier transport and recombination mechanisms. As *N*_D_ increases, the internal electric field is strengthened due to the increased ionized charge density in the space charge region. This stronger field promotes more efficient separation of photogenerated electron–hole pairs and reduces their recombination probability. This reduction in recombination is clearly manifested by an increase in the recombination resistance (*R*_rec_), as observed in the impedance diagrams, indicating that recombination losses become less dominant at high doping levels.

The decrease in the recombination rate has a direct impact on the *V*_oc_. Indeed, increasing *R*_rec_ is accompanied by a reduction in the reverse saturation current *J*_0_, which leads to an increase in *V*_oc_. Thus, higher *N*_D_ values contribute to improved separation of quasi-Fermi levels and increased maximum voltage supplied by the cell. Furthermore, FF also benefits from the increase in *N*_D_. The strengthening of the internal field and the reduction of recombination losses allow for more efficient carrier extraction under forward bias, which improves the linearity of the *J*–*V* curve around the maximum power point. Consequently, the FF increases with *N*_D_, reflecting an overall improvement in the electrical quality of the junction.

## 4 Electrical behavior of Al–ZnO/i-ZnO/CdS/SnS structure at temperature 300 K

### 4.1 Current voltage simulation at various SnS absorber thickness

**Fig. 8:**
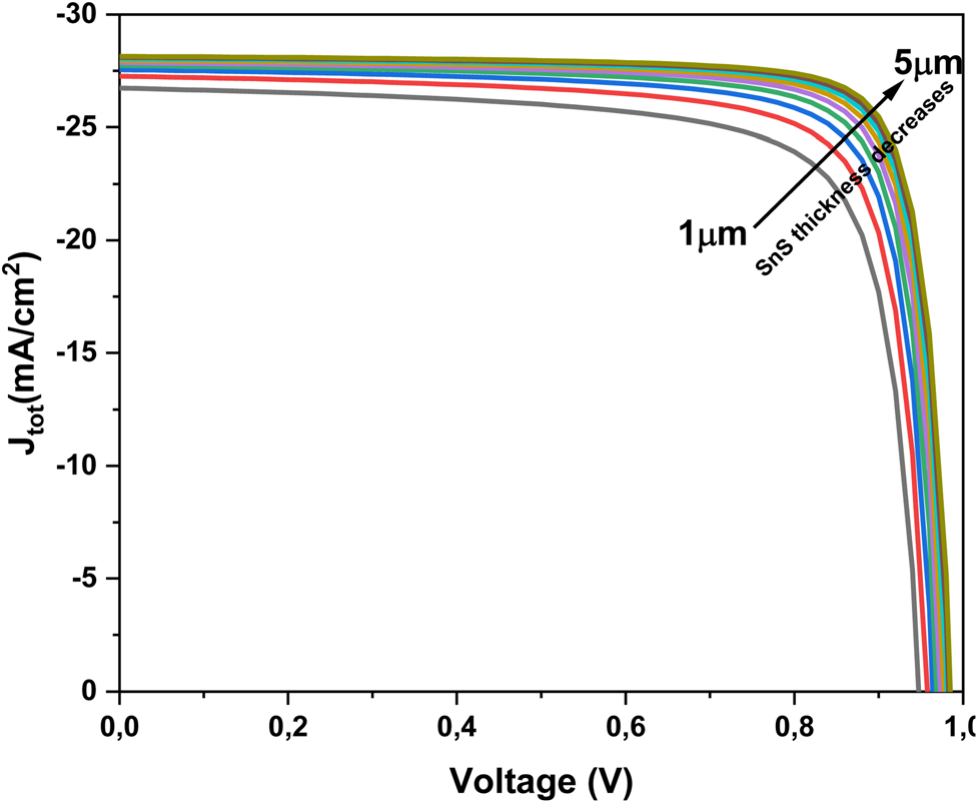
*J*–*V* curves of Al–ZnO/i-ZnO/CdS/SnS solar cells measured at various SnS absorber thicknesses.

The thickness of the absorber layer is a critical design parameter in thin-film solar cells, as it directly influences light absorption, carrier generation, and recombination dynamics. To better understand its impact on device behavior, *J*–*V* simulations were performed for Al–ZnO/i-ZnO/CdS/SnS solar cells at different SnS thicknesses. The *J*–*V* curves presented in Fig. 8 demonstrate the effect of SnS absorber thickness on device performance. As the thickness increases from 1 µm to 5 µm, the short-circuit current density (*J*_sc_) improves significantly, owing to enhanced optical absorption and higher carrier generation. However, this improvement is accompanied by a slight decrease in *V*_oc_, which can be attributed to stronger recombination and resistive losses in thicker layers. These results indicate the existence of an optimum absorber thickness, beyond which additional material does not substantially improve performance and may even reduce efficiency.

**Fig. 9:**
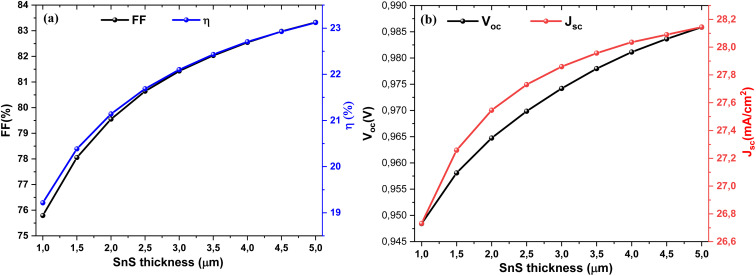
Variation of (a) FF and *η*, (b) *V*_oc_ and *J*_sc_ parameters of the solar cells as a function of SnS thickness.

Fig. 8 depict the effect of SnS absorber thickness on the photovoltaic performance of the Al–ZnO/i-ZnO/CdS/SnS solar cell. As shown in Fig. 9a, both the FF and efficiency (*η*) increase steadily with thickness. FF rises from ∼76% at 1 µm to approximately 83% at 5 µm, while *η* improves from ∼19% to ∼23% over the same range. This improvement is attributed to enhanced optical absorption, which generates a higher density of photo-generated carriers. Fig. 9b shows the evolution of *V*_oc_ and *J*_sc_ with thickness. The *J*_sc_ increases markedly from ∼26.7 to ∼28.2 mA cm^−2^ as the thickness grows, reflecting better light harvesting and carrier generation. *V*_oc_ also rises slightly (from ∼0.945 to ∼0.985 V), which can be linked to reduced recombination losses and improved junction quality in thicker absorbers. These results confirm that increasing the SnS thickness enhances device performance, particularly in the 3–4 µm range, where a balance between photon absorption and carrier transport is achieved. Beyond this range, further gains become less pronounced due to increased recombination and resistive effects.

### 4.2 Capacitance–voltage frequency analysis

**Fig. 10:**
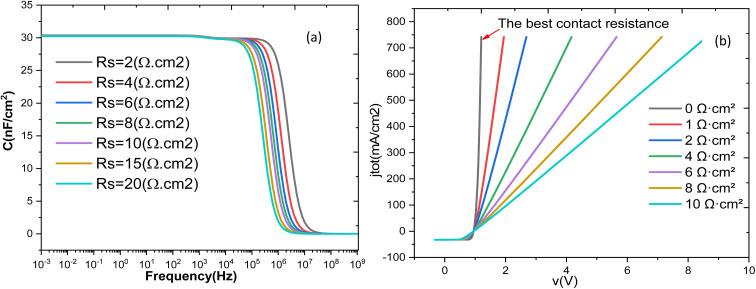
(a) Variation of cell capacitance as a function of frequency (b) *J*_total_ as a function of voltage for all simulation resistors.

In this study, *C*–*V* simulations were carried out both for the complete device and for a simplified one-diode p-CZTS/n-CdS junction model. In the p–n junction model, the i-ZnO and Al: ZnO layers were excluded, and the back contact was treated under the flat-band approximation to eliminate any secondary diode effects. To minimize the influence of deep-level defects on the apparent doping profile, the *C*–*V* simulations were performed at high frequency (1 MHz). Deep-level traps can alter the space charge density and the depletion width; however, at high frequencies, these defects are unable to follow the AC signal. Consequently, a reliable carrier concentration profile can only be obtained at high frequencies and low temperatures, where the deep-level traps are effectively frozen out. Fig. 10 presents the capacitance–frequency response of the Al–ZnO/i-ZnO/CdS/SnS solar cell. In the capacitance plot, a high and nearly constant capacitance value is observed in the low-frequency region (CBF), corresponding to full charge response from the depletion region. As frequency increases, capacitance decreases sharply toward the high frequency limit (CHF), where carriers cannot follow the applied AC signal. The equivalence capacitance identified at the transition provides an estimate of interface-related processes.

The *C*–*V* characteristics are essential for determining the apparent doping profiles, the built-in junction voltage, and the width of the space-charge region. Recent studies on CIGS solar cells have shown that ultra-thin passivation layers significantly modify the measured doping profile obtained from *C*–*V* analysis, particularly through interactions with passivated front contacts.^54^ Moreover, the impact of selective contacts on capacitance measurements in perovskite solar cells has been highlighted, requiring more sophisticated models to correctly interpret the *C*–*V* profile.^55^

### 4.3 Complex impedance spectrum simulation at various SnS absorber thickness

**Fig. 11:**
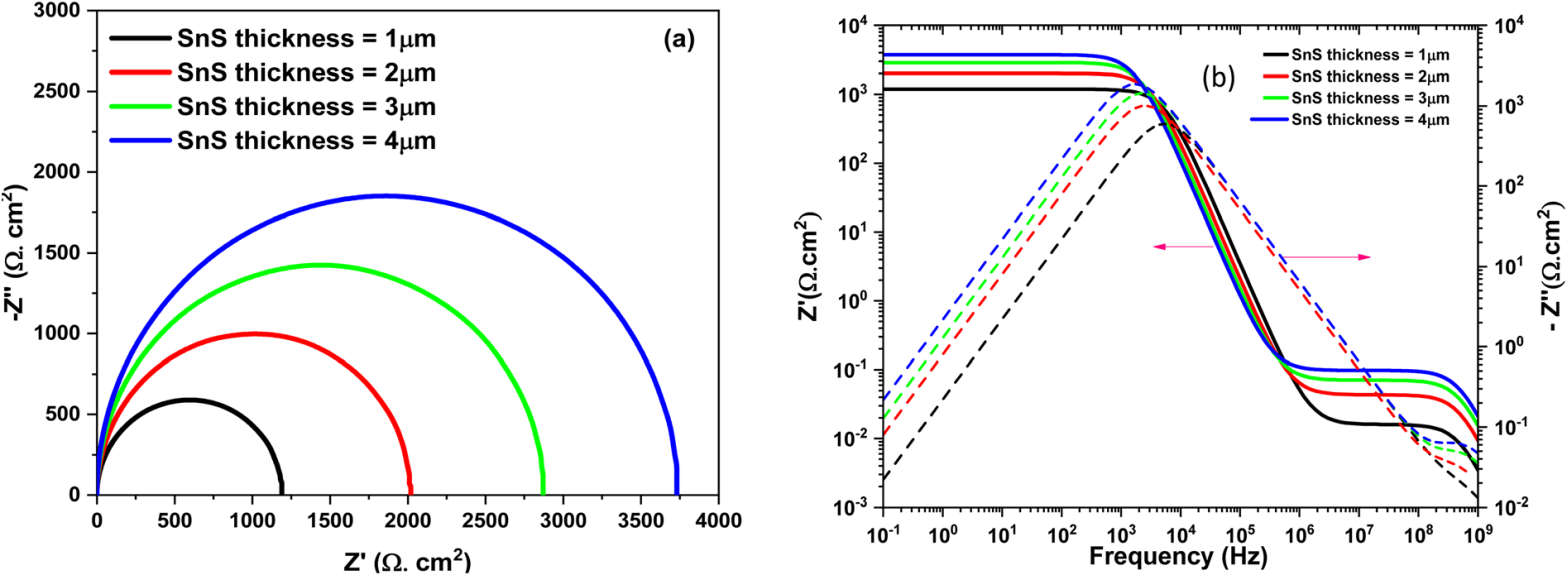
The complex impedance spectrum of the solar cells structure at different SnS thickness. (a) Nyquist plot, (b) Bode plot.

Fig. 11 shows the effect of SnS thickness on impedance spectrum. This effect results in an increase in the cell's resistance, which affects the solar cell efficiency by causing a drop in the *V*_oc_.^56^ Increasing the thickness of the SnS film (likely a semiconductor material used in the solar cell) leads to an increase in the internal resistance of the cell.^57^ A higher resistance in the cell can slow down the charge transport processes (electrons and holes), which may limit the cell's performance, particularly the *V*_oc_. The *V*_oc_ is a crucial parameter in solar cells, representing the maximum voltage the cell can produce when no current is flowing.^1^ A decrease in *V*_oc_ suggests that the increase in internal resistance prevents the cell from reaching its maximum voltage potential, thus reducing the overall efficiency of the solar cell.^58^

### 4.4 Monitoring the operation of the solar cell *via* the equivalent circuit

**Fig. 12:**
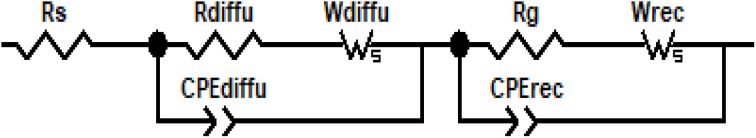
Electrical equivalent circuit models used to fit the impedance spectra.

Using the equivalent electrical circuit (Fig. 12), and the deconvolution technique, one can determine the electrical circuit that models the impedance spectra specific to each interface. The complex impedance of the circuit modeled by two blocs in parallel and the resistor series is flows.

These results (Tables 4 and 5) demonstrate that the electrical response of the device is governed by both bulk-related and interface-driven relaxation mechanisms, underscoring the complex interplay between charge transport and recombination in the Al–ZnO/i-ZnO/CdS/SnS solar cell. The Zview software simulation (Table 4) shows that *R*_s_ remains almost constant with SnS thickness, while *R*_1_ increases and *C*_dl_ decreases as shown in Table 4, reflecting stronger recombination and reduced charge accumulation in thicker absorbers. In contrast, *τ*_rec_, *T*_1-rec_, *W*_rec_, and *F*_r-rec_ remain nearly unchanged, indicating stable relaxation dynamics. Thus, absorber thickness mainly affects recombination resistance and capacitance without altering the fundamental transport mechanisms.^59^

**Table 4:** Parameters obtained Z-view simulation during the change of SnS thickness for interface recombination

Layer	*R*_s_ (µΩ cm^2^)	Interface – diffusion/transport of loads						
SnS thickness (µm)		*R*_1_ (Ω cm^2^)	*C*_dl_1__ (nF)	*T*_1_ (ns)	*τ*_diffu_ (ns)	*W*_1_ (GΩ s^1/2^)	*F*_r_1__ (GHz)	CPE1-T (nF) (CPE-p = 1)
1	0.195	0.016	19.21	0.307	0.307	3.26	0.519	19.20
2		0.043	7.01	0.304	0.304	3.29	0.524	7.01
3		0.070	4.29	0.303	0.330	3.30	0.525	4.29
4		0.098	3.10	0.304	0.304	3.26	0.524	3.10

**Table 5:** Parameters obtained Z-view simulation during the change of SnS thickness for interface diffusion

Interface – recombinassions p–n						
*R*_2_ (kΩ)	*C*_dl_2__ (µF)	*T*_2_ (s)	*τ*_rec_ (ms)	W_2_ (kΩ s^1/2^)	*F*_r_2__ (kHz)	CPE_2_-T (nF), CPE-p = 1
1.19	24.68	2.94	29.37	30.41	5.42	24.7
2.02	24.71	4.99	49.90	20.0	3.19	24.7
2.87	24.72	7.10	70.98	14.1	2.24	24.7
3.73	24.76	9.24	92.40	10.8	1.72	24.8

**Fig. 13:**
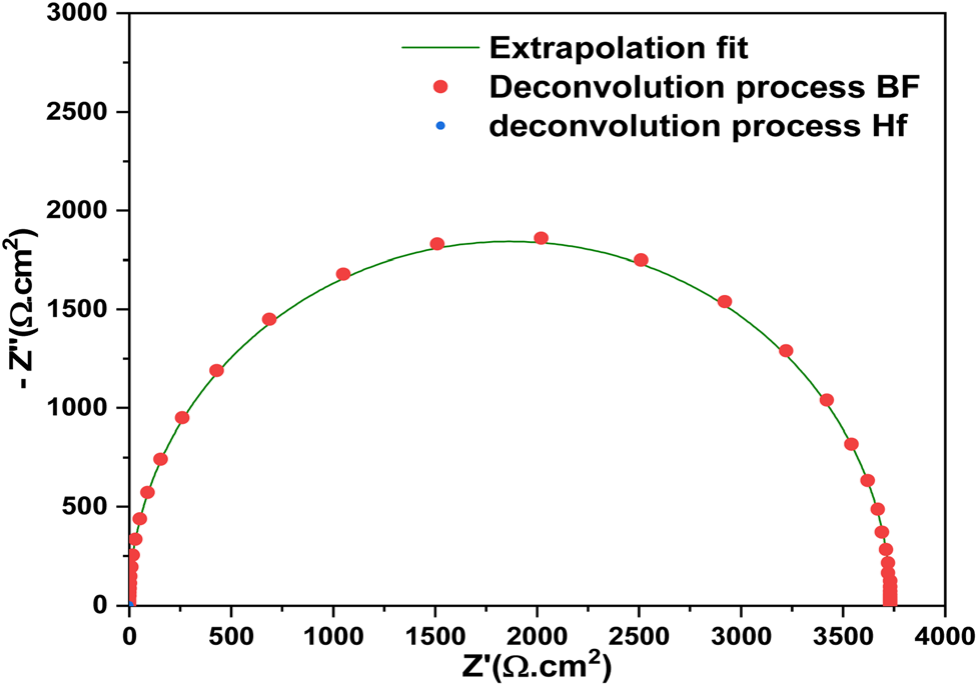
Nyquist diagram showing masked or unresolved processes within the cell.

The Nyquist plot of the Al–ZnO/i-ZnO/CdS/SnS solar cell was accurately fitted using extrapolation, confirming the validity of the equivalent circuit (Fig. 13). Deconvolution analysis reveals two main contributions: a bulk frequency (BF) process related to charge transport and recombination at the CdS/SnS interface, and a high frequency (HF) process associated with interfacial polarization in the ZnO layers. This separation provides deeper insight into the mechanisms governing carrier dynamics and device impedance behavior.

**Fig. 14:**
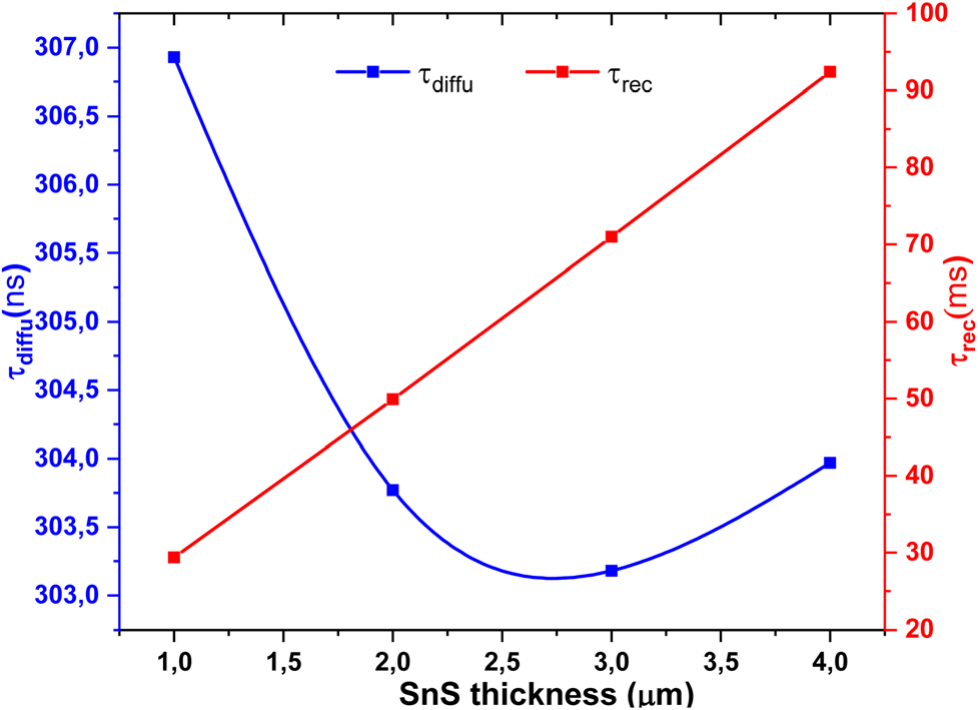
Variation of relaxation times (*τ*_diffu_ and *τ*_rec_) as a function of SnS absorber thickness.

Fig. 14 shows the variation of relaxation times *τ*_rec_ and *τ*_diffu_ with SnS absorber thickness. *τ*_diff_, associated with interfacial charge transfer, decreases with thickness up to ∼3 µm before slightly increasing, while *τ*_rec_, linked to bulk recombination, rises steadily. This opposite trend highlights the trade-off between interfacial dynamics and bulk transport in optimizing SnS thickness for efficient solar cell operation.^60^

## 5 Effect of *R*_s_ for SnS absorber thickness = 4 µm, *T* = 300 K

### 5.1 Current voltage simulation at various *R*_s_ values

**Fig. 15:**
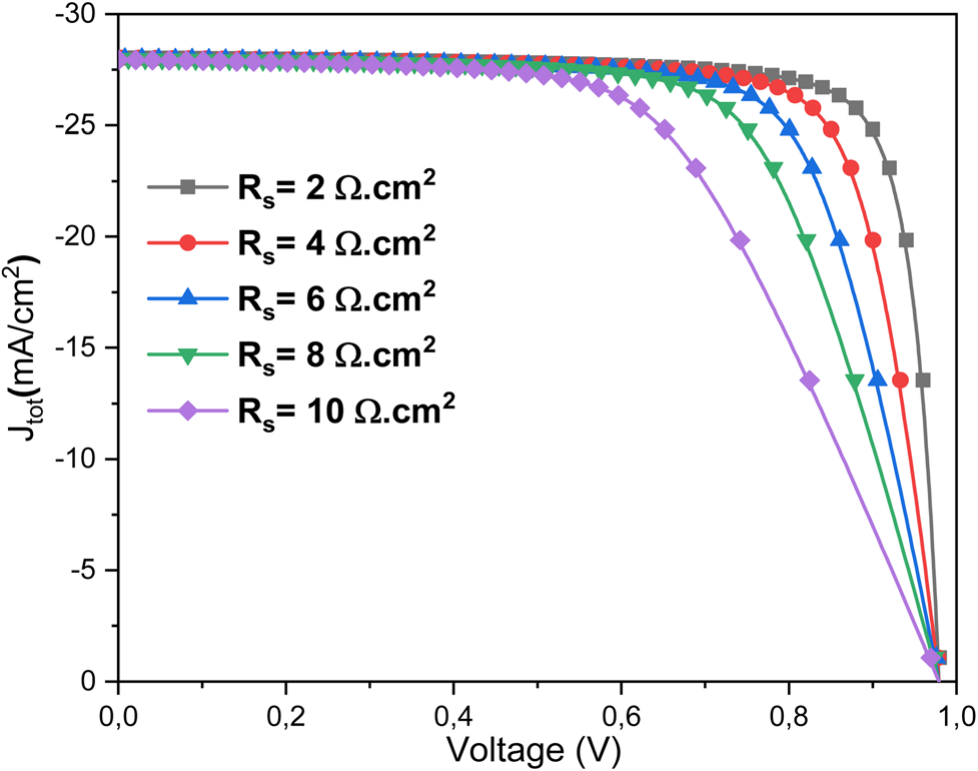
Current density *versus* voltage measurements of solar cells at different *R*_s_ values.

Fig. 15 depicts the effect of *R*_s_ on the *J*–*V* characteristics of the Al–ZnO/i-ZnO/CdS/SnS solar cell. At low *R*_s_ (2 Ω cm^2^), the device exhibits sharp diode-like behavior, with high FF and efficiency. As *R*_s_ increases progressively from 4 to 10 Ω cm^2^, the slope of the *J*–*V* curve near the maximum power point decreases, reflecting enhanced resistive losses that lower both the FF (%) and power conversion efficiency *η*. The *J*_sc_ remains nearly constant, as *R*_s_ primarily affects carrier extraction rather than light absorption. However, *V*_oc_ shows a slight reduction at higher *R*_s_ values due to increased recombination and reduced carrier collection efficiency. These results highlight the critical importance of minimizing *R*_s_ through improved electrode contact quality and optimized layer conductivity to maximize the photovoltaic performance of Al–ZnO/i-ZnO/CdS/SnS solar cells.^61^ Resistance *R*_s_ is a key electrical parameter that encompasses all internal resistances to current flow in a solar cell: those of the metal contacts, semiconductor layers, interfaces, and connections. Although unavoidable, this resistance must be minimized to ensure good cell performance. A high *R*_s_ cause is ohmic loss during current flow, resulting in a drop in output voltage, particularly at the maximum power point (MPP).^62^ Consequently, this directly reduces the power delivered and therefore the overall efficiency of the cell. The higher the current (especially under strong sunlight), the more significant this loss is.

Regarding quantum efficiency, *R*_s_ does not directly affect the number of absorbed photons or the generation of electron–hole pairs, but it does affect the cell's ability to efficiently extract the generated charges. If *R*_s_ is too high, the generated carriers may recombine before being collected, which reduces the output current, particularly in the external quantum efficiency (EQE) measured at different illumination levels.^63^ Thus, to achieve excellent efficiency and high quantum efficiency, it is crucial to reduce the *R*_s_ as much as possible. This is achieved by optimizing conductive materials, layer thickness, interface quality, and metallization.

**Fig. 16:**
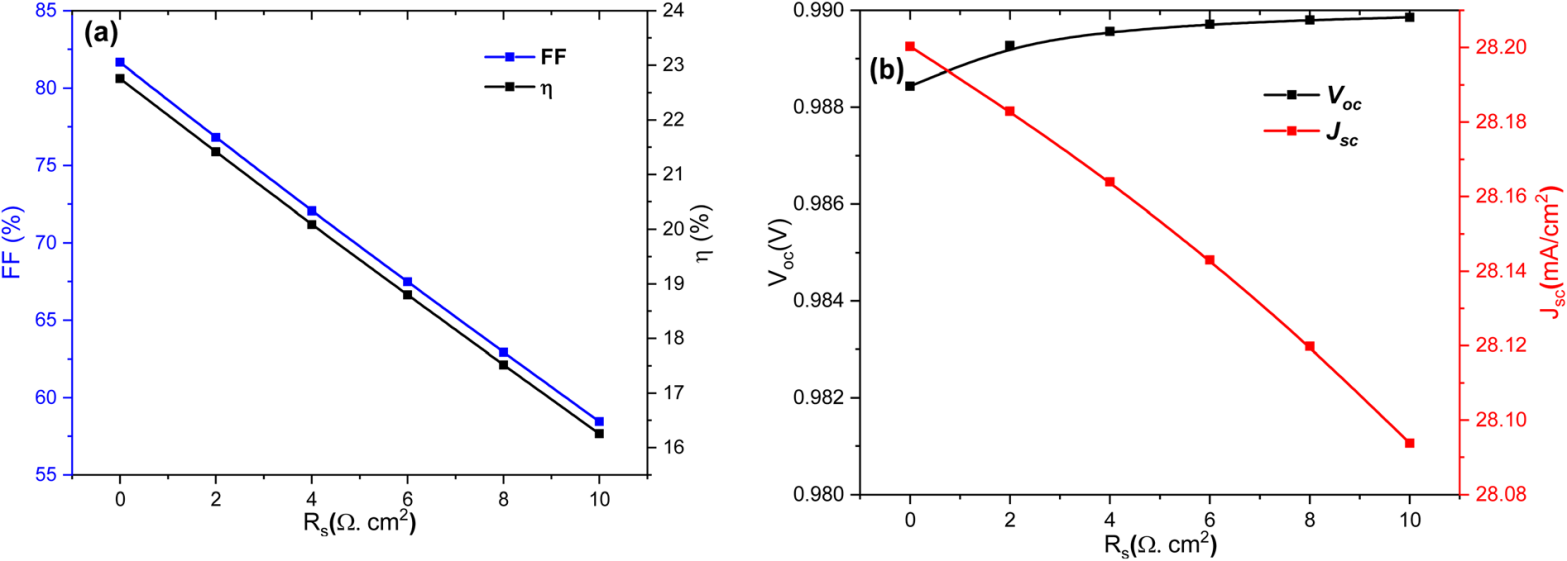
Dependence of (a) FF and *η*, and (b) *V*_oc_ and *J*_sc_ of Al–ZnO/i-ZnO/CdS/SnS solar cells on the *R*_s_.

Fig. 16a presents the strong dependence of the FF and power conversion efficiency *η* on the *R*_s_. Both parameters decrease almost linearly as *R*_s_ increases, indicating that resistive losses critically degrade charge extraction efficiency. For instance, when *R*_s_ rises from 0 to 10 Ω cm^2^, the FF drops from ∼82% to ∼60%, and *η* decreases from ∼22% to below 17%. This emphasizes the crucial role of maintaining low *R*_s_ to ensure optimal device performance. Fig. 16b shows the evolution of *V*_oc_, *J*_sc_ with *R*_s_. The *V*_oc_ remains nearly unchanged, with only a negligible decline, reflecting its weak sensitivity to *R*_s_. In contrast, *J*_sc_ decreases gradually from ∼28.04 to ∼27.94 mA cm^−2^ as *R*_s_ increases, suggesting that resistive losses hinder efficient carrier collection. This confirms the dominant effect of *R*_s_ is on FF and efficiency.

Table 6 presents the effect of *R*_s_ on solar cell performance. An increase in this resistance acts as an additional barrier to the efficient transport of charge carriers, thereby limiting their collection at the electrodes. This constraint results in a more pronounced internal voltage drop, which reduces the available output current. Consequently, the FF decreases, leading to a reduction in the maximum extractable power of the cell. This degradation directly impacts the overall efficiency, confirming that photovoltaic performance is highly sensitive to the value of *R*_s_. It follows that minimizing this parameter is a key factor in the optimization and design of high-efficiency solar devices.

**Table 6:** Al–ZnO/i-ZnO/CdS/SnS solar cells performances parameters at different *R*_s_ values

*R*_s_ (Ω cm^2^)	*V*_oc_ (V)	*J*_sc_ (mA cm^−2^)	FF (%)	*η* (%)
0	0.9811	28.0358	82.5449	22.7056
2	0.9813	28.0201	77.6780	21.3587
4	0.9813	28.0029	72.8681	20.0247
6	0.9813	27.9839	68.1079	18.7042
8	0.9813	27.9628	63.5682	17.4445
10	0.9813	27.9392	59.0215	16.1832

### 5.2 Complex impedance spectrum simulation various *R*_s_ values

**Fig. 17:**
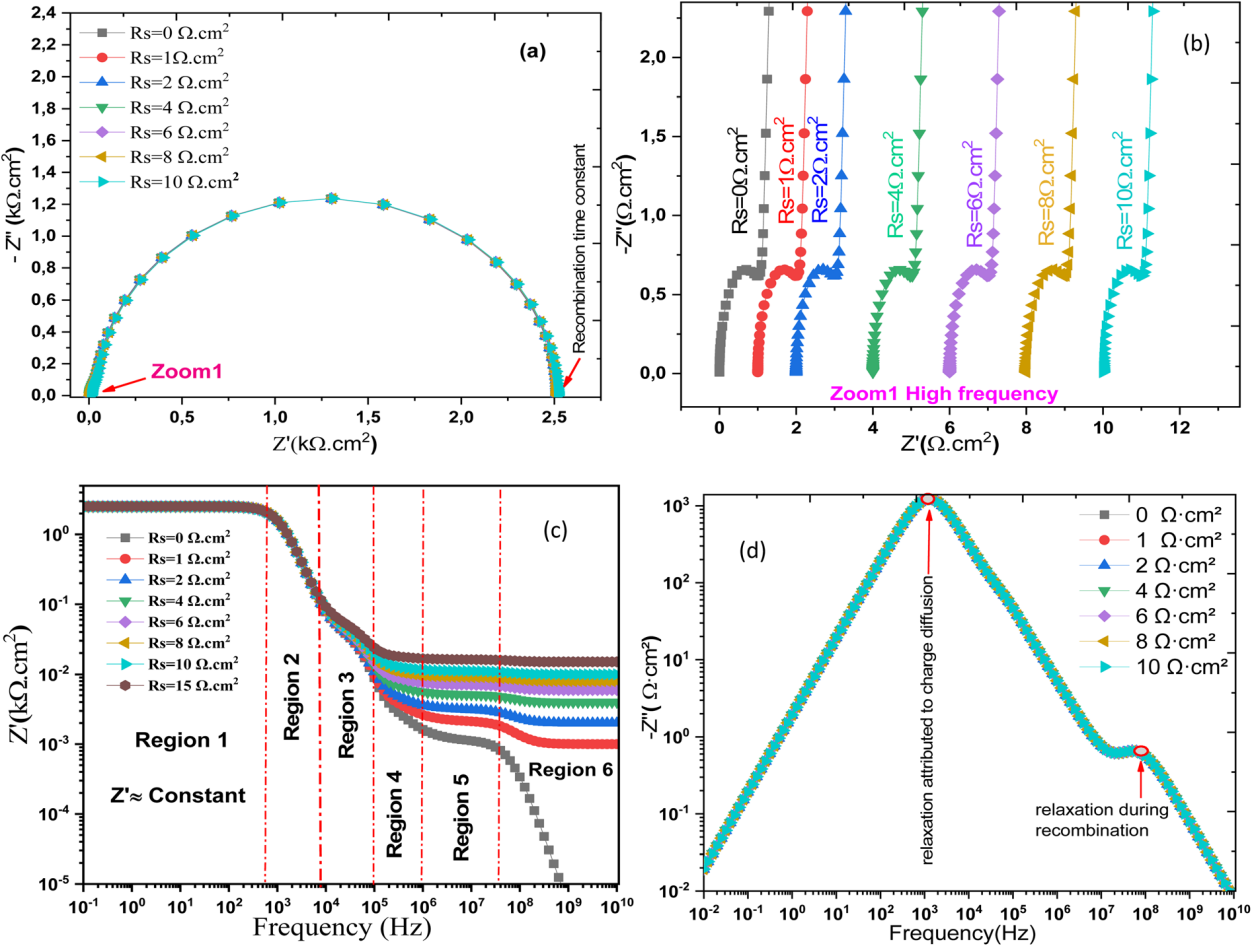
Nyquist diagram (a and b) and (c and d) *Z*″ and *Z*′ frequency dependence of Al–ZnO/i-ZnO/CdS/SnS solar cell structure.

Fig. 17a presents the Nyquist diagrams of the Al–ZnO/i-ZnO/CdS/SnS solar cells for different *R*_s_ values (*R*_s_). The increase of *R*_s_ leads to a larger semicircular arc, reflecting higher recombination resistance and energy dissipation within the device. Zoom 1 (Fig. 17b) highlights the systematic broadening of the curves with *R*_s_, confirming the detrimental effect of resistive losses on charge transport. Fig. 17c and d depicts the variation of the real (*Z*′) and imaginary (*Z*″) parts of the impedance as a function of frequency. At low *R*_s_, the relaxation peak appears sharper and occurs at higher frequency, corresponding to faster charge transport and reduced recombination.^64^ As *R*_s_ increases, the relaxation peak shifts to lower frequencies and broadens significantly, indicating slower carrier dynamics and stronger resistive constraints. This further confirms the frequency shift and the correlation between *R*_s_ and interfacial relaxation behavior structure at different *R*_s_ values.

**Fig. 18:**
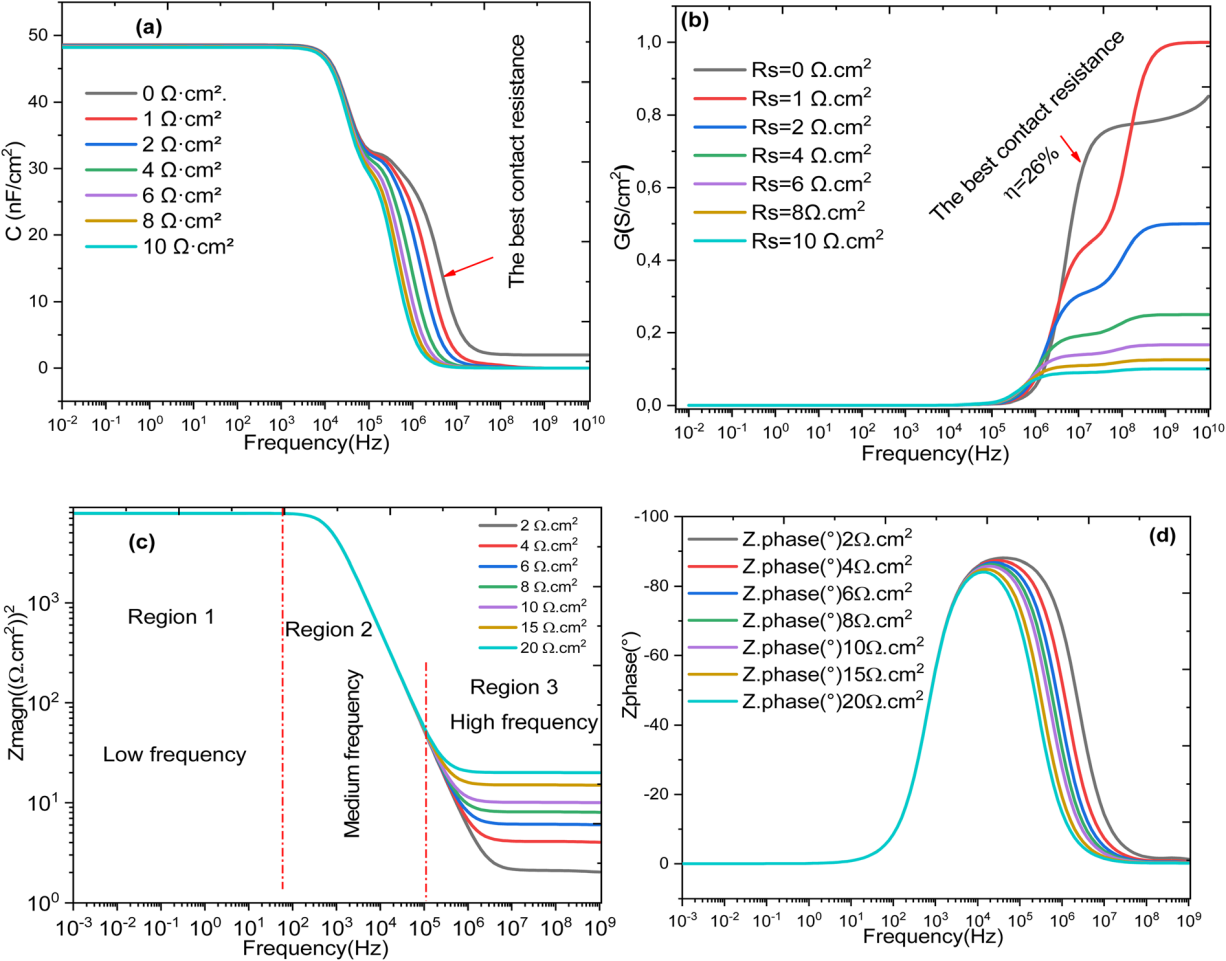
Evolution of the frequency-dependent (a) capacitance, (b) conductance, (c) electric modulus, and (d) loss angle influenced by *R*_s_.

Fig. 18 shows the effect of *R*_s_ on the main electrical parameters of the solar cell as a function of frequency: capacitance (a), conductance (b), electrical modulus (c), and loss angle (d). It is clearly observed that increasing the *R*_s_ negatively affects all these parameters, indicating a progressive degradation of the cell's performance. Complementarily, the transparent conductive oxide (TCO) contribution (*R*_TCO_) is determined by its sheet resistance and geometry and affects lateral carrier transport toward the contacts. High TCO resistance increases voltage losses and reduces the fill factor (FF). The absorber resistance (*R*_absorber_) depends on the bulk resistivity and thickness of the active layer. Materials with low mobility or high resistivity enhance this contribution, limiting charge transport and further reducing the FF. The contact resistance (*R*_contact_) arises from the quality of the metal/semiconductor and heterojunction interfaces. Poorly optimized contacts can significantly increase *R*_s_, leading to a larger voltage drop under load and decreased FF. At low frequencies, the capacitance significantly decreases with higher *R*_s_, suggesting reduced charge accumulation at the interfaces. Conductance follows a similar trend reflecting increased limitations in charge transport. The electrical modulus (|*Z*|) generally increases with *R*_s_, indicating a greater opposition to AC current flow. Finally, the loss angle increases over certain frequency ranges, reflecting enhanced energy dissipation within the device.^65^ These results confirm that *R*_s_ is a major limiting factor, affecting both charge transport and storage phenomena in the cell, particularly in the absorber and interface regions.^66^ Frequency-domain analysis thus provides valuable insight into its overall impact on the electrochemical response of the device.

**Fig. 19:**
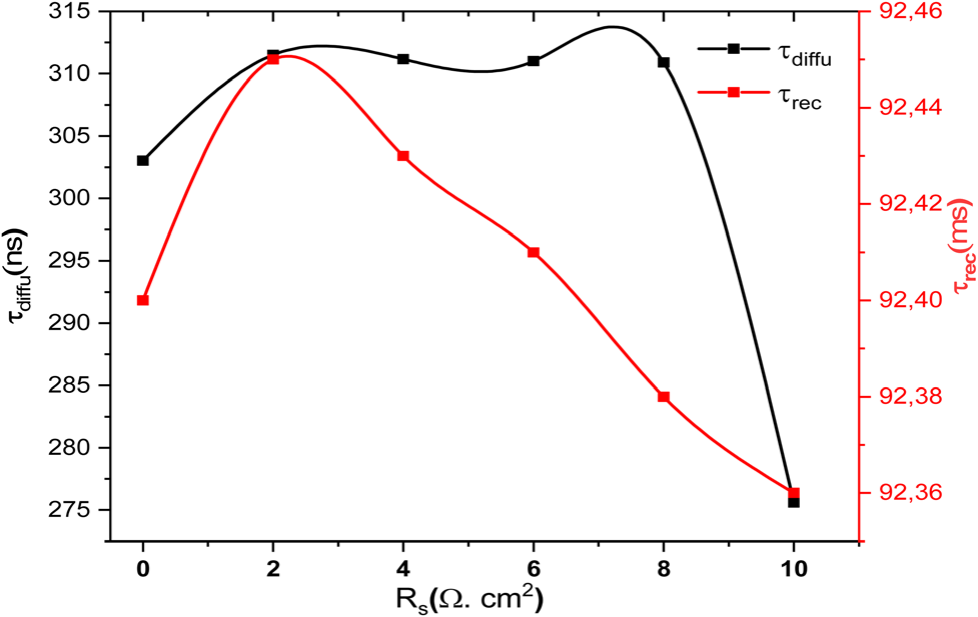
Evolution of relaxation times (*τ*_diff_, *τ*_rec_) as a function of *R*_s_ in Al–ZnO/i-ZnO/CdS/SnS solar cells.

Fig. 19 compares the evolution of the relaxation times *τ*_rec_ and *τ*_diffu_ as a function of series *R*_s_. The diffusion-related relaxation time *τ*_diff_ (ns range) shows moderate variations: it increases slightly up to *R*_s_ ≈ 6–7 Ω cm^2^ before dropping sharply at *R*_s_ = 10 Ω cm^2^. By contrast, the recombination-related relaxation time *τ*_rec_ (ms range) remains almost constant, with only a slight decrease as *R*_s_ increases. This stability suggests that recombination at the CdS/SnS junction is largely unaffected by *R*_s_, confirming that *R*_s_ mainly influences charge transport rather than junction recombination.^67^ Overall, *τ*_rec_ is more sensitive to *R*_s_ than *τ*_diffu_, showing that resistive effects dominate in the diffusion pathway, while recombination dynamics at the absorber/buffer interface remain stable.

*The Analysis of high-frequency (HF) behavior* – the high-frequency relaxation time *τ*_HF_ remains near constant as *R*_s_ increases up to ∼7–8 Ω cm^2^, then a sharp drop in *τ*_HF_ is observed for *R*_s_ > 8 Ω cm^2^. The small variation in *τ*_HF_ at low *R*_s_ indicates a localized process weakly influenced by serial transport, demonstrating behavior typical of interfacial recombination and thermally activated deep traps. This result is consistent with the activation energy *E*_a_HF__ = 0.47 eV. This shows that this energy corresponds to an interfacial activation barrier, relatively insensitive to *R*_s_.

*Analysis of low-frequency (LF) behavior –* the mid-frequency relaxation time *τ*_BF_ decreases continuously with increasing *R*_s_, with no stable plateau. A process strongly coupled to transport is observed, closely linked to charge accumulation, a signature of shallow traps and trapping/untrapping.

## 6 Effect of temperature for SnS absorber thickness = 4 µm

### 6.1 Current voltage chacteristics at different temperature

**Fig. 20:**
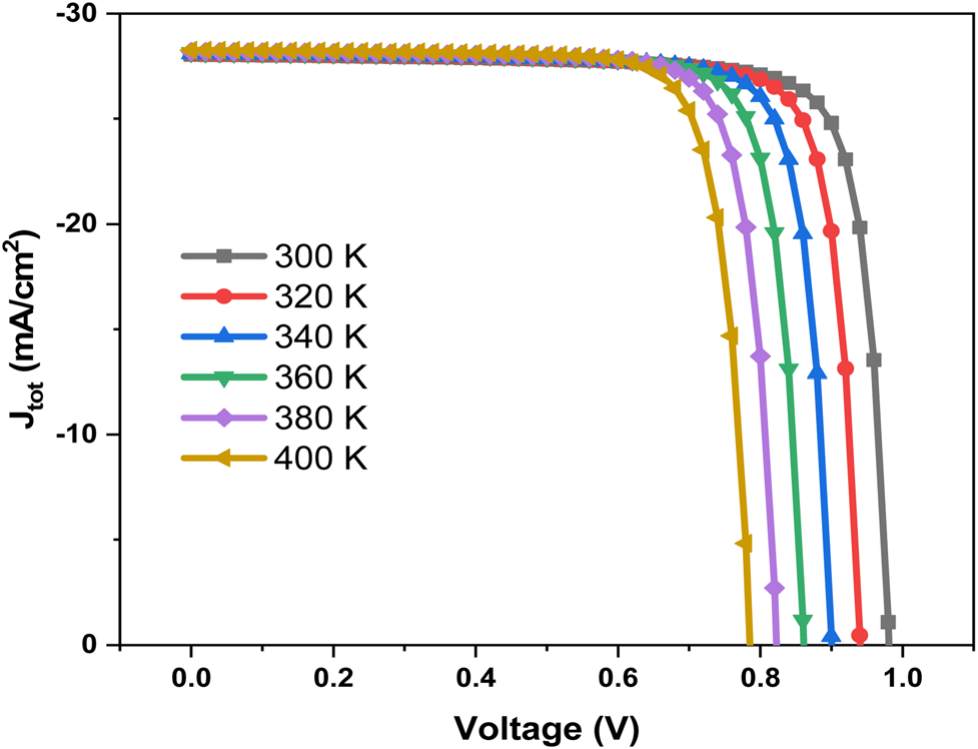
Current density *versus* voltage measurements of Al–ZnO/i-ZnO/CdS/SnS solar cells at different temperature.

Fig. 20 shows the *J*–*V* characteristics of the solar cell measured at temperatures ranging from 300 K to 400 K. As temperature increases, the slope of the *J*–*V* curve near *V*_oc_ decreases, indicating higher recombination losses and reduced charge collection efficiency. The overall photovoltaic performance degrades with temperature, mainly due to the reduction in *V*_oc_ and FF. Fig. 20a quantifies these variations by plotting FF and *η* as a function of temperature. Both parameters decrease steadily with temperature rise, with *η* dropping from above 22% at 300 K to below 19% at 400 K. This decline reflects the detrimental influence of thermally activated recombination and increased *R*_s_.

**Fig. 21:**
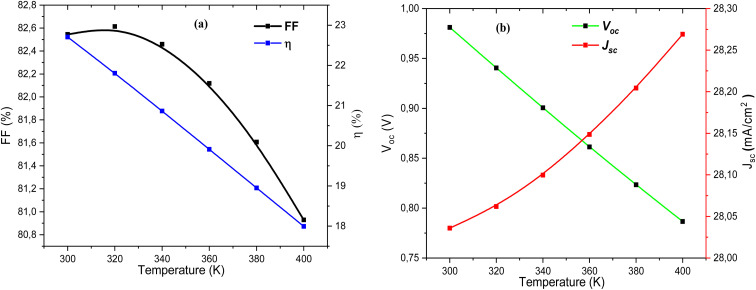
Variation of (a) FF (%) and eta (%), and (b) *V*_oc_ and *J*_sc_ parameters of Al–ZnO/i-ZnO/CdS/SnS solar cells as a function of temperature.

Fig. 21a quantifies these variations by plotting FF and *η* as a function of temperature. Both parameters decrease steadily with temperature rise, with *η* dropping from above 22% at 300 K to below 19% at 400 K. This decline reflects the detrimental influence of thermally activated recombination and increased *R*_s_. Fig. 21b illustrates the opposing behavior of *V*_oc_ and *J*_sc_.

The *V*_oc_ decreases monotonically with increasing temperature, consistent with the narrowing of the bandgap and enhanced recombination. In contrast, *J*_sc_ shows a slight increase (from ∼28.0 to ∼28.3 mA cm^−2^) due to enhanced carrier excitation at higher temperatures. However, this gain is insufficient to compensate for the losses in *V*_oc_ and FF, resulting in a net decrease in efficiency. The obtained results confirm that temperature rise negatively affects the device performance, primarily through reduced *V*_oc_ and FF, despite the small improvement in *J*_sc_. Thermal control and interface optimization are therefore critical to stabilize the efficiency of SnS-based solar cells under operating conditions.^68^

### 6.2 Complex impedance spectrum simulation at different temperatures

**Fig. 22:**
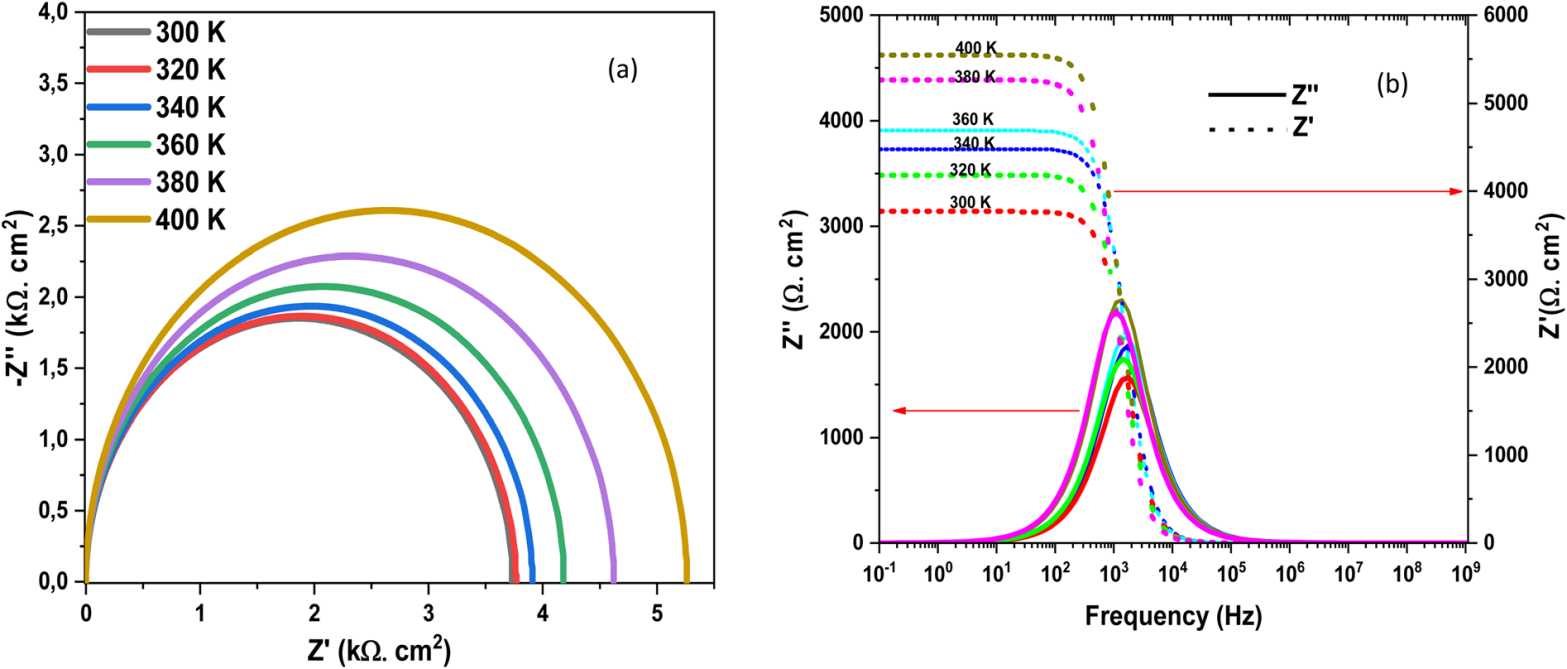
Nyquist diagram and (a) and (b) *Z*″ and *Z*′ dependence of frequency of Al–ZnO/i-ZnO/CdS/SnS solar cells structure at different temperature.

Fig. 22a presents the Nyquist diagrams at different temperatures (300–400 K). The semicircle diameter increases systematically with temperature, reflecting a rise in charge-transfer resistance and recombination losses within the device.^69^ At higher temperatures (380–400 K), the impedance response becomes significantly larger, highlighting the detrimental impact of thermal activation on carrier transport. Fig. 22b shows the frequency dependence of the real (*Z*′) and imaginary (*Z*″) components of the impedance.

**Fig. 23:**
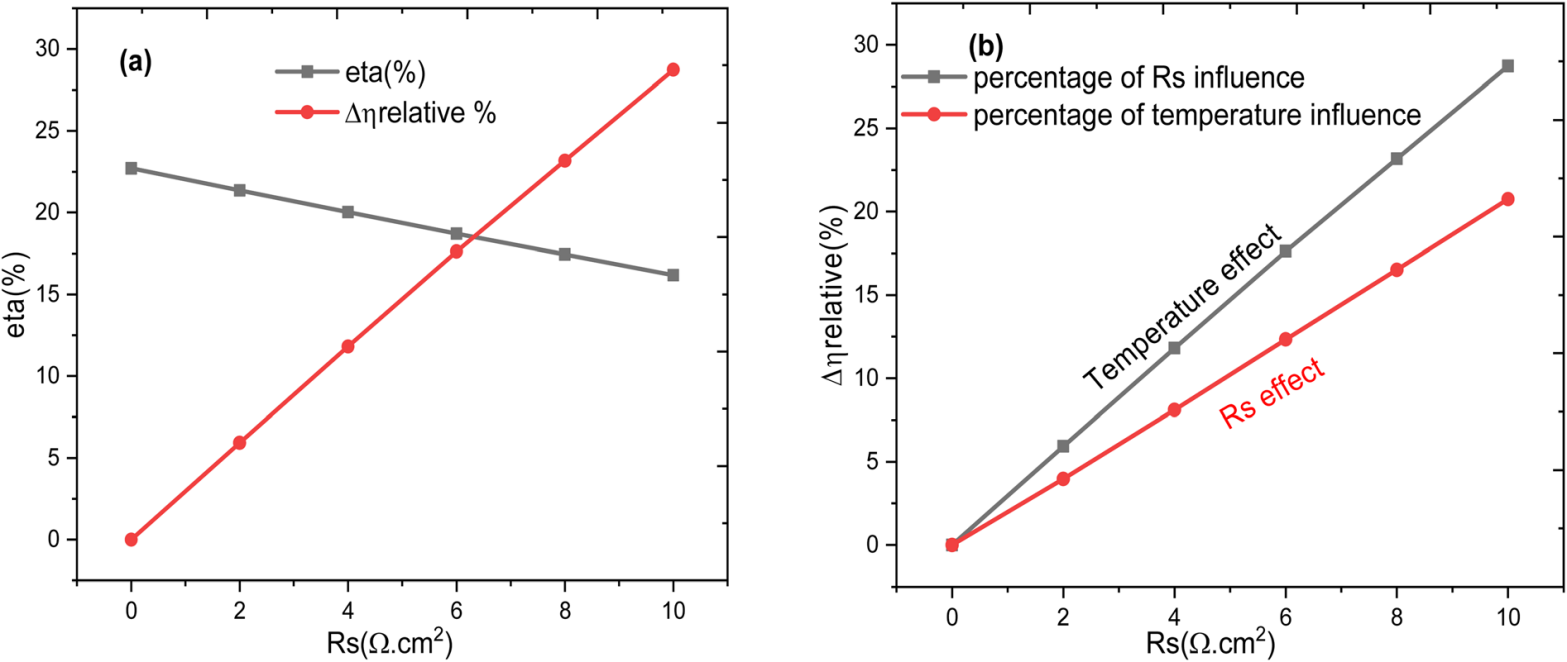
Variation of efficiency and effect of *R*_s_ (a) and (b) variation of the relative percentage of efficiency for temperature effect with *R*_s_.

At low temperatures, the relaxation peak of *Z*″ occurs at higher frequencies and with smaller amplitude, indicating faster charge transport and reduced recombination. For the high-frequency response (*τ*_1_), which is mainly attributed to bulk transport and bulk recombination in the absorber, increasing temperature enhances carrier mobility and thermally activates carriers out of shallow traps. As a result, the bulk resistance decreases with temperature, leading to a reduction of *τ*_1_. This behaviour is consistent with thermally activated charge transport and reduced bulk recombination lifetime at elevated temperatures. In contrast, the low-frequency response (*τ*_2_) is dominated by interfacial charge accumulation and defect-assisted recombination at the heterojunction (*e.g.*, CdS/SnS or CdS/ZnO). With increasing temperature, more interface states become active, and carrier capture/emission rates at these defects increase. This promotes stronger interfacial recombination and larger interfacial capacitance due to enhanced charge storage, while the associated recombination resistance decreases more slowly or may even increase depending on the barrier properties. Consequently, the product *R*_ct_ × *C*_int_ increases, giving rise to an increase of *τ*_2_ with temperature. Physically, this opposite trend indicates that *τ*_1_ is governed by thermally activated bulk transport (faster at higher *T*), whereas *τ*_2_ reflects interface-dominated processes where temperature enhances carrier trapping, accumulation, and recombination dynamics at the junction.^70^ These results confirm that temperature rise degrades the impedance response of the device. Therefore, effective thermal management and interface passivation are essential strategies to preserve the performance of Al–ZnO/i-ZnO/CdS/SnS solar cells under real operating conditions. Fig. 23 represents the evolution of the efficiency and the effect of the *R*_s_ as a function of the *R*_s_ for an absorber thickness of 4 µm and a temperature of 300 K. It represents also the evolution of the temperature effect on the efficiency as a function of temperature and as a function of *R*_s_ for a thickness of 4 µm and *R*_s_ = 1 Ω cm^2^.

From the two curves shown in Fig. 23, we can observe that the *R*_s_ effect (red curve) causes a greater voltage drop than the temperature effect (black curve), especially at high currents. Therefore, in this plot, the *R*_s_ effect is more detrimental to efficiency than the temperature effect.^71^ Temperature affects the entire cell and modifies the physical characteristics of the material; it can have a more severe cumulative impact, especially in the long term. The resistance *R*_s_ is a technological parameter that can be minimized during design.

In recent years, researchers have devoted considerable efforts to improving processing, interface, and buffer limitations, particularly recombination at the CdS/SnS interface.^72^ Most experimental efforts have emphasized that annealing temperature, deposition routes, and buffer architecture influence recombination at the interface and the formation of the n-CdS/p-SnS secondary phase.^73,74^ For example, Fathy and colleagues^73^ achieved a power conversion efficiency of 4.2% under optimized electrodeposition conditions. The authors attributed this lower performance to experimental challenges in reducing contact/buffer losses and reducing defect density. To mitigate this, improved buffer layers such as ZnO or ZnS have been proposed to increase FF and efficiencies of solar heterojunctions.^75^ Alternative buffer layers such as ZnS and Zn(O,S) strongly affect interfacial recombination and band alignment at the absorber/buffer junction.^76^ By modifying the conduction band offset, these layers control carrier accumulation and recombination at the interface. A small positive offset suppresses interface recombination, while a negative offset enhances it. Improved band alignment and lower interface defect density increase the interfacial recombination resistance (*R*_ct_) and reduce interfacial capacitance, indicating weaker trap-assisted recombination and less charge accumulation. Zn(O,S) is particularly advantageous because its band alignment can be tuned *via* composition,^76^ while ZnS and In_2_S_3_ generally provide better interface passivation than CdS.^77^ Different from the optimization of transparent conductive oxides (i-ZnO, AZO), the use of low-resistivity back electrodes, such as graphite, has been proposed to stabalize current extraction and boost FF.^78^ Passivation layers such as Al_2_O_3_ and SiO_2_ have been used to protect the device from oxidation and reduce surface traps.^79^ In addition, physical encapsulants such as glass or polymer laminates prevent material degradation caused by oxygen and moisture ingress. As presented in Table 7, careful buffer engineering, a cell with configuration COMSOL 1D simulation (TiO_2_/SnS/BFO/spiro-OMeTAD), optimized buffer thickness yield an optimum efficiency of 23.3% and a remarkable FF exceeding 80%.^80^ Experimental studies by Islam *et al.*^72^ on the SnS/CdS device reported an efficiency of approximately 5% due to higher recombination rates and potentially poor interface quality. Hybrid structures have also demonstrated moderate improvements in efficiency (approximately 8.5%), indicating the potential of SnS as an effective hole-transport layer in multi-junction devices. This study reports robust optimal performance (*η* = 22.76%, FF = 81.65%, *V*_oc_ = 0.98 V, and *J*_sc_ = 28.20 mA cm^−2^), highlighting the impact of absorber thickness optimization and *R*_s_ minimization.

**Table 7:** Experimental and theoretical comparison of SnS/CdS heterojunction solar cell performance from literature

Device architecture	Method	*V*_oc_ (V)	*J*_sc_ (mA cm^−2^)	*η* (%)	PCE (%)	Ref.
SnS/CdS heterojunction	Simulation	0.98	28.20	81.65	22.76	This study
Glass/ITO/n-CdS/p-SnS/Mo	Experimental	1.00	13.6	0.32	4.35	73
CdS/ZnO/ITO thin films	Experimental	0.226	19.64	34	1.5	80
TiO_2_/SnS/BFO/spiro-OMeTAD	Simulation	0.917	31.045	81.627	23.245	80
SnS/CZTS/TiO_2_/ITO	Experimental	1.09	>32	>87	>30	81

### 6.3 Activation energy

The electrical conductivity associated with diffusion and recombination mechanisms follows the following expressions eqn (4) and (5). In order to determine the activation energies, the diffusion phenomenon is associated with high-frequency processes, while the bulk recombination phenomenon corresponds to medium-frequency processes. Based on the evolution of relaxation times obtained by impedance spectroscopy modeling, it is possible to determine two activation energies.4
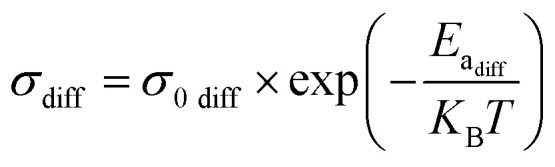
5
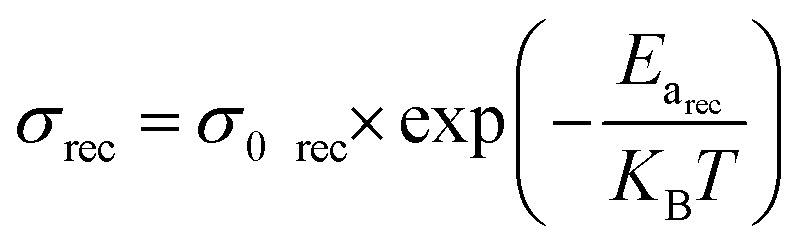


**Fig. 24:**
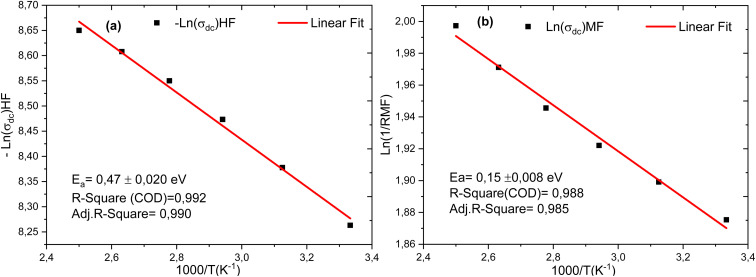
(a) and (b) illustrate, respectively, the evolution of the activation energy at high and low frequencies of the cell structure.

The effect of temperature on open circuit voltage (*V*_oc_) can be analysed using eqn (6).6
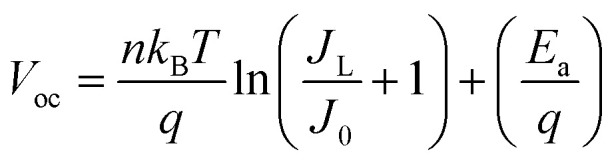
where, *E*_a_ is the activation energy of dominant recombination, *n* is the diode ideality factor, *J*_0_ is the reverse saturation current prefactor, *J*_L_ is photocurrent, *k*_B_ is the Boltzmann constant, *T* is temperature, and *q* is the elementary charge. To observe the dominant recombination effect in both the solar cells, temperature dependent *V*_oc_ have been investigated in the range of 300–400 K and presented with extrapolation to 0 K. The extrapolations of *V*_oc_ to 0 K intercept at 1.56 eV for SnS solar cell. Fig. 24a and b, respectively, illustrate the evolution of the activation energy associated with high-frequency and medium-frequency processes for the proposed architecture. The activation energy extracted from the temperature dependence of *V*_oc_ reflects the dominant volumetric recombination mechanism and it is close to the absorber's effective band gap. In contrast, impedance spectroscopy reveals lower activation energies associated with high- and mid-frequency processes. The high-frequency activation energy (0.47 eV) is attributed to trap-assisted interfacial recombination, while the mid-frequency activation energy (0.15 eV) is attributed to charge relaxation induced by shallow traps. The difference between these energies highlights the ability of electrochemical impedance spectroscopy (EIS) to resolve localized and kinetically distinct loss mechanisms not accounted for by *V*_oc_ (*T*) analysis.

## 7 Conclusions

In this work, p-SnS/n-CdS heterojunction solar cells with Al–ZnO/i-ZnO window layers were analyzed through SCAPS-D device simulator and impedance spectroscopy. The study confirmed that absorber thickness, *R*_s_, and operating temperature are key parameters governing photovoltaic performance. An optimized SnS thickness of 4 µm enabled an efficiency of 22.76% and *V*_oc_ of 0.77 V under standard conditions of 1.5G AM and one sun at 1000 W m^−2^. *R*_s_ was shown to primarily degrade the FF and efficiency, while temperature increase reduced *V*_oc_ and FF. Nonetheless, a slight improvement in *J*_sc_ was noted. The influence of boundary conditions reveal that device efficiency is particularly sensitive to the back-contact work function and interface recombination velocity. A reduction in the back-contact work function or an increase in SRV enhances interfacial recombination, which largely lowers *V*_oc_ and FF due to increased carrier losses at the contact. In contrast, the front contact primarily affects carrier extraction and *R*_s_, thus moderately influencing FF and *J*_sc_. Impedance analysis revealed two distinct relaxation mechanisms: bulk recombination at the CdS/SnS interface and interfacial polarization in the ZnO layers. Moreover, the interface-specific behavior of *τ*_1_ and *τ*_2_ under thermal stress highlighted the contrasting roles of ZnO/CdS and CdS/SnS junctions in device stability. These findings confirm that with proper interface engineering and thermal regulation, SnS-based heterojunctions can evolve into a competitive and sustainable alternative for the next-generation thin-film solar cells. Impedance spectroscopy was used to assess cell efficiency and accurately model polarization and diffusion mechanisms. Deconvolution analysis distinguished the different processes according to their frequency domain: low, medium, and high frequency. Conduction phenomena, particularly hopping and diffusion, were studied in detail to better understand their respective contributions to the overall electrochemical response of the cell under study. Further, this study demonstrated that complex impedance spectroscopy is a powerful and indispensable tool for the in-depth analysis of solar cells, as it provides access to charge transport and recombination mechanisms that cannot be resolved by steady-state *J*–*V* measurements alone. By separating the electrical response into distinct frequency domains, impedance analysis enables the identification of multiple relaxation processes associated with bulk recombination, interfacial trap-assisted recombination, and charge accumulation effects. The extraction of frequency-dependent activation energies and relaxation times offers direct insight into the kinetic and energetic nature of these loss mechanisms. Importantly, the strong correlation between impedance-derived parameters, temperature-dependent *V*_oc_, and device performance metrics (FF and *R*_s_) highlights the unique capability of impedance spectroscopy to link microscopic defect physics with macroscopic device behavior. Consequently, complex impedance analysis emerges as a crucial diagnostic tool for guiding material optimization, interface engineering, and performance improvement in thin-film solar cells.

## Consent for publication

This article has the consent of all the authors.

## Conflicts of interest

The authors have no competing interests.

## Data Availability

The data associated with the findings of this study are available from the corresponding author upon reasonable request.
